# Classic Maya Bloodletting and the Cultural Evolution of Religious Rituals: Quantifying Patterns of Variation in Hieroglyphic Texts

**DOI:** 10.1371/journal.pone.0107982

**Published:** 2014-09-25

**Authors:** Jessica Munson, Viviana Amati, Mark Collard, Martha J. Macri

**Affiliations:** 1 Department of Linguistics, University of California Davis, Davis, California, United States of America; 2 Department of Computer & Information Science, University of Konstanz, Konstanz, Germany; 3 Human Evolutionary Studies Program and Department of Archaeology, Simon Fraser University, Burnaby, British Columbia, Canada; 4 Department of Archaeology, University of Aberdeen, St Mary’s Building, Aberdeen, Scotland, United Kingdom; 5 Department of Native American Studies, University of California Davis, Davis, California, United States of America; University of Bern, Switzerland

## Abstract

Religious rituals that are painful or highly stressful are hypothesized to be costly signs of commitment essential for the evolution of complex society. Yet few studies have investigated how such extreme ritual practices were culturally transmitted in past societies. Here, we report the first study to analyze temporal and spatial variation in bloodletting rituals recorded in Classic Maya (ca. 250–900 CE) hieroglyphic texts. We also identify the sociopolitical contexts most closely associated with these ancient recorded rituals. Sampling an extensive record of 2,480 hieroglyphic texts, this study identifies every recorded instance of the logographic sign for the word *ch’ahb’* that is associated with ritual bloodletting. We show that documented rituals exhibit low frequency whose occurrence cannot be predicted by spatial location. Conversely, network ties better capture the distribution of bloodletting rituals across the southern Maya region. Our results indicate that bloodletting rituals by Maya nobles were not uniformly recorded, but were typically documented in association with antagonistic statements and may have signaled royal commitments among connected polities.

*No longer can evolutionists dismiss such works* [of ritual sacrifice] *as scholarly, but irrelevant, esoterica–explorations of the bizarre but insignificant elements of Precolumbian culture…we must address this ideological data base with the same interest that we would give to studies of settlement patterns, subsistence systems, or political institutions* [1].

## Introduction

Ritual acts of blood sacrifice are dominant themes in Pre-Columbian art and archaeology, and are an essential topic for understanding the development of early complex societies in Mesoamerica, as Arthur Demarest suggested over 30 years ago. Sociologists and anthropologists have long made similar arguments that intense rituals enhance social cohesion and promote cooperation for human society [2]–[4]. These so-called “costly ritual behaviors”–and the traces they leave in the archaeological and historical records–are therefore worthy subjects of scientific inquiry that require explanations derived from a unified theoretical framework [5]. Although many scholars recognize that sacrificial rituals involving the dedication of human lives and blood to supernatural beings were essential to the religious foundation and political order of Mesoamerican societies [6]–[8], cultural evolutionary explanations for these behaviors are not featured prominently due to earlier monolithic views of religious change [1]. Rather than assume that blood rituals form part of a universal Mesoamerican religion, we focus our attention on the distribution and spread of bloodletting rituals recorded in Classic Maya hieroglyphic texts. We examine this specific class of sacrificial practices because selected examples of Classic Maya bloodletting have been used to illustrate how these extravagant and costly displays increased the level of commitment to dominant ideology in past complex societies [9], [10]. With these considerations in mind, our aim is threefold: 1) to analyze the temporal and spatial variation of bloodletting rituals in the Maya hieroglyphic record, 2) to examine factors associated with the uneven distribution of these bloodletting records, and 3) to demonstrate how current cultural evolutionary models can provide key insights on the historical dynamics of past cultural practices.

### Blood sacrifice in ancient Mesoamerica

Abundant archaeological and historical evidence supports the prevalence of religious practices involving blood sacrifice throughout Mesoamerica before the arrival of the Spanish [11]–[13]. These include a variety of ritual behaviors involving non-lethal forms of bloodletting and body piercings, as well as deadly practices of human and animal sacrifice, heart extraction, and decapitation. Among the most noted examples, Aztec human sacrifice stands out for its ritual violence and bloodshed. Performed in the religious precincts of Tenochitlan, ritual sacrifice was a primary instrument for social integration and political legitimacy that intersected with militaristic and marketplace practices, as well as with beliefs about the cosmological order [6]. Although human sacrifice was arguably less common in ancient Maya society, physical evidence indicates that offerings of infant sacrifices and other rituals involving decapitation were important religious practices during the Classic period [14], [15]. Scenes of personal bloodletting and mutilated captives further illustrate the range of sacrificial rituals within Classic Maya society [16], [17]. While material remains suggest sacrificial rites may have conferred political power for the ruling class [7], bloodletting rituals were not the exclusive domain of elites. Ethnohistorical documents indicate these practices were pervasive among most social classes in Mesoamerica [18]. While blood sacrifice may represent a set of loosely shared religious beliefs for many ancient Mesoamerican people, the codes of conduct that regulated these and other cultural practices are dependent upon significant social and historical factors [19]. To avoid monolithic portrayals of these ritual practices, such specific details deserve greater analytic attention to understand the mechanisms and contexts in which blood rituals were practiced and spread through ancient Mesoamerican societies.

**Figure 1:**
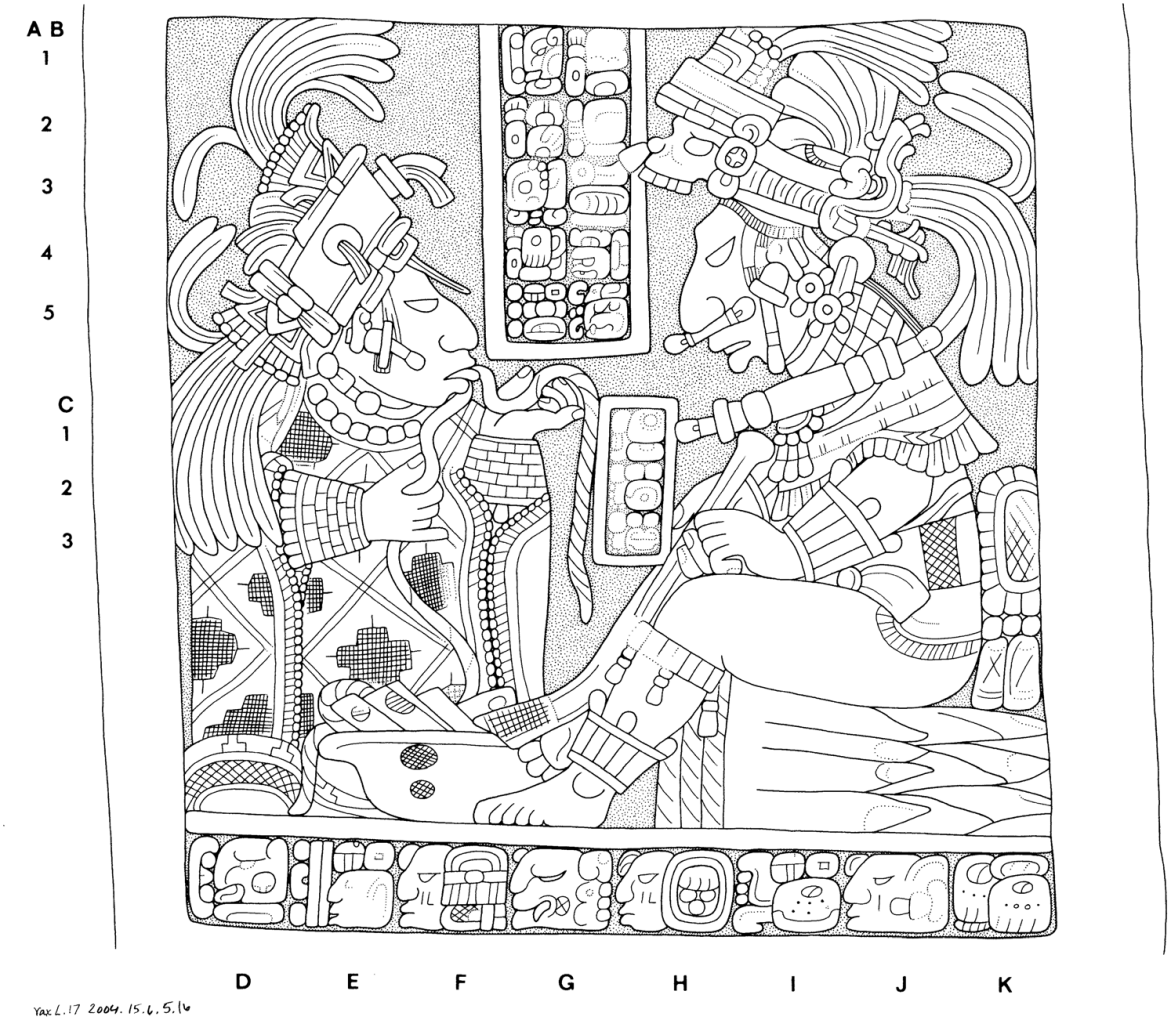
Bloodletting scene from Yaxchilan Lintel 17. This image of Classic Maya bloodletting depicts a royal man using a stingray spine to pierce his penis and a noble woman pulling a thorny vine through her tongue in ritual acts of self-sacrifice. Drawing by Ian Graham [81], Yaxchilan, Lintel 17. © President and Fellows of Harvard University, Peabody Museum of Archaeology and Ethnology, 2004.15.6.5.16. Digital file #101240031.

**Figure 2:**
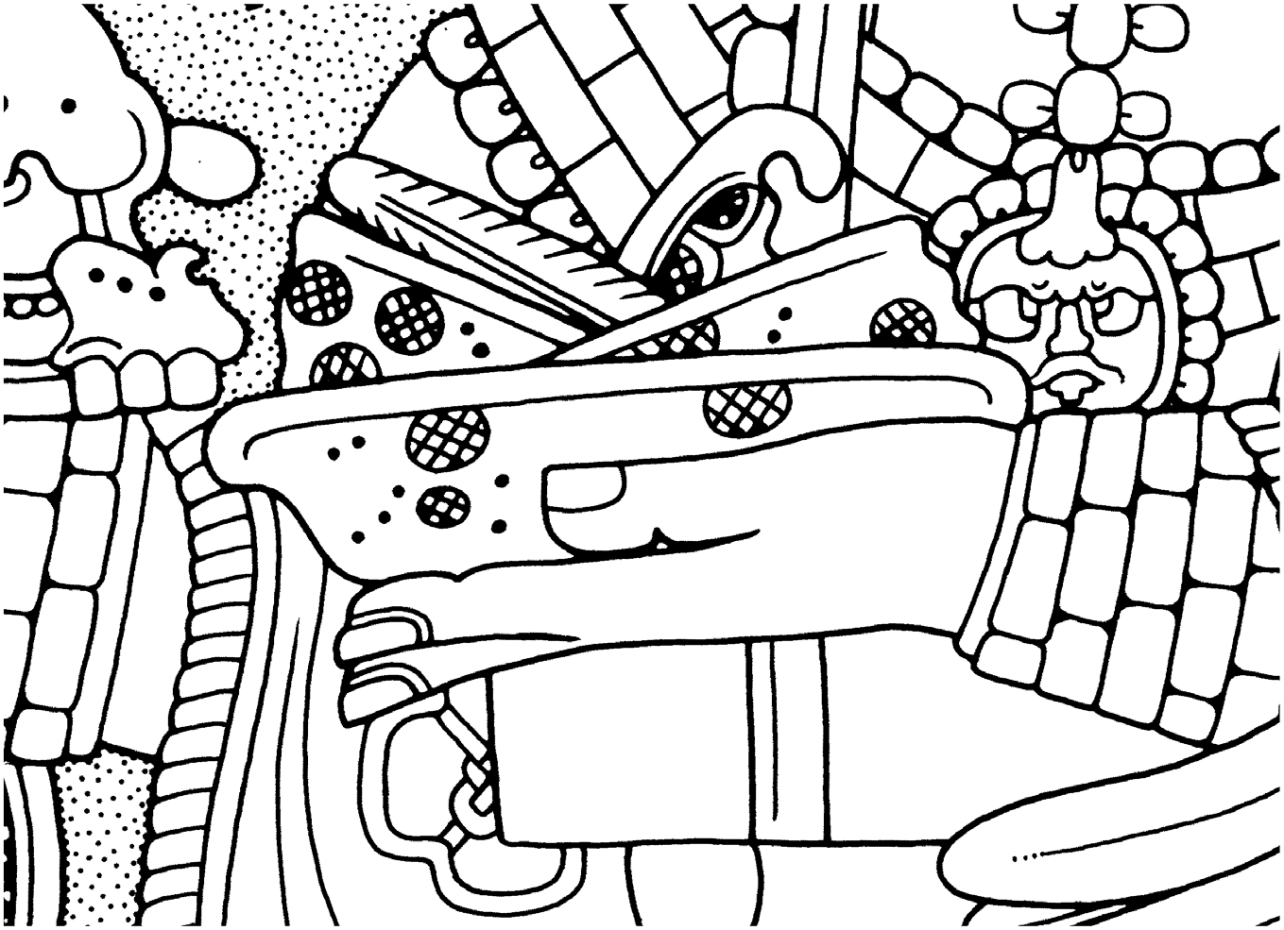
Bowl containing instruments used in Classic Maya bloodletting rituals. From Yaxchilan Lintel 25. Detail of drawing by Ian Graham [81], Yaxchilan, Lintel 25, front edge. © President and Fellows of Harvard University, Peabody Museum of Archaeology and Ethnology, 2004.15.6.5.22. Digital file #101240034.

Classic Maya bloodletting is a well-documented ancient ritual based on iconographic and epigraphic data as well as biological studies on the methods and risks associated with these practices [8], [20]–[24]. Bloodletting rituals involved puncturing or perforating different body parts using a variety of instruments. Classic period iconographic and figurative representations show individuals piercing their tongue or penis to withdraw blood in acts of personal sacrifice (Figure 1). Sacrificial scenes found in Postclassic codices also include images of penis perforation and bloodletting from the ears, along with other representations of human sacrifice that have been associated with the Maya calendar [25]. Some of the objects depicted in these bloodletting scenes include: bowls with lancets, stingray spines, obsidian and flint blades, bone awls, cord, and bark paper, many of which have also been recovered archaeologically (Figure 2). Ethnohistorical sources, including the *Relación* by Diego de Landa [26] further corroborate these pictorial scenes of bloodletting:

“They offered sacrifices of their own blood, sometimes cutting themselves around in pieces and they left them in this way as a sign. Other times they pierced their cheeks, at other times their lower lips. Sometimes they scarify certain parts of their bodies, at others they pierced their tongues in a slanting direction from side to side and passed bits of straw through the holes with horrible suffering, other slit the superfluous part of the virile member leaving it as they did their ears.” [27]

For the Classic period, images of bloodletting carved in stone monuments and painted on pottery illustrate elaborate and complex rituals performed by Maya royalty and supernatural figures. These scenes depict important dynastic rituals, which served various purposes associated with agricultural ceremonies, ancestor communication, metaphorical acts of procreation, as well as other royal obligations [8], [20], [21], [28]–[31]. Although Maya bloodletting rituals can be included in a broader category of Mesoamerican religious practices, earlier studies overlook two key issues for the cultural evolution of religion: 1) the extent to which the frequency of these rituals varied through space and time and 2) the contexts and potential mechanisms that contributed to the spread of these ritual practices in Classic Maya society.

Despite significant advances in the decipherment of Classic Maya writing over the last 40 years, incomplete and imprecise database records have hampered quantitative studies of the occurrence of these rituals in the historical record. Here we present evidence for ritual bloodletting drawn from the Maya Hieroglyphic Database (MHD), a unique set of digitized hieroglyphic records, in order to characterize the intensity and transmission of bloodletting rituals recorded on Maya stelae. These deciphered texts include detailed social and political information about Maya rulers, toponyms for important places and landscape features, as well as calendar dates referring to specific ritual events. In addition, many of these hieroglyphic inscriptions are found on stone monuments in the same location where they were originally placed. Unlike the colonial documentation of Aztec human sacrifice, Classic Maya hieroglyphic texts can be considered a unique example of “indigenous” written testimony [32]. Still, it is important to keep in mind that these data are not objective descriptions of historical events. Rather, they are reflections of how the Maya perceived, remembered, and chose to record selected aspects of their religious practice.

### Costly signals and religious behavior

Social scientists have long speculated that intense rituals generate collective benefits that offset individual costs by reinforcing social bonds and promoting group solidarity [2], [3]. Recent evolutionary approaches to religion formalize these ideas by offering possible models to interpret the spread of costly displays and their consequences for human society [33], [34]. These approaches suggest that cooperation among large groups of genetically unrelated individuals can be facilitated by extravagant behaviors that reliably signal commitments to social groups and shared ideals. Hard-to-fake religious behaviors are argued to promote beliefs in moralizing supernatural agents by enhancing within-group interpersonal trust through low monitoring costs and by stabilizing prosocial norms [34]. For example, results from one recent study illustrate how both ritual performers and observers of a Hindu body-piercing ritual were more likely to make charitable donations to the local temple than performers of a low-ordeal ritual [35]. Increasingly, natural experiments and economic games demonstrate the behavioral impacts of costly rituals in contemporary religious populations [36], [37]. However, we are not aware of any other study that addresses how such extravagant rituals varied over time or spread between different social groups in the past–essential information for assessing the significance of religion for cultural evolution.

Using the framework of signaling theory [38], inscribed stone monuments located in the central plazas of ancient Maya cities can be interpreted as important transmitters of cultural information. Fraser Neiman [39] originally introduced this evolutionary principle to Mesoamerican archaeology by arguing that Maya stelae constitute reliable, although wasteful, advertisements of a ruler’s ability to outcompete his political neighbors. In Neiman’s study, the construction of dated monuments was treated as a lavish investment of resources, which would have otherwise been used for bodily maintenance and reproduction. While the construction of monuments may involve costly displays of symbolic capital [38], bloodletting may present significant health risks, including inflammation and pain, tissue necrosis, as well as reduced or impaired fertilization which impose real fitness costs [23], [40]. Some even propose that such practices of genital mutilation may have functioned as costly signals to counteract potential free-rider problems in times of war [41]. However, for the spread of ritual practices and religious beliefs, Henrich points out that such extreme costs are not necessarily required for effective cultural transmission [9]. Under the right circumstances, costly acts such as ritual bloodletting can be interpreted as “credibility enhancing displays” (CREDs) if they reliably signal an individual’s commitment or professed beliefs [9], [10].

The representation and recording of bloodletting rituals on public monuments sends powerful, if not conspicuous, messages about the status and role of Classic Maya rulers and their progeny. Despite the documented risks associated with bloodletting [23], ritual acts of blood sacrifice are traditionally regarded as a central duty for divine Maya kings. Current scholarship argues these were necessary rites of passage that legitimated an individual’s right to rule in Classic Maya society [21], [29], [30]. In the words of David Stuart, “an important function of monumental art, through the evocation of bloodletting, [was] to express the legitimacy and the metaphysical validity of the office of divine king” [21]. Such conclusive statements appear compatible with the hypothesized function of CREDs presented in Henrich’s model [9], as well as other signaling models in which displays serve to justify and perpetuate inequalities in power, prestige and access to resources [4], [42]. In this paper, we apply this signaling perspective to examine specific ritual information recorded on Maya hieroglyphic monuments and test current hypotheses about the function and spread of these ritual practices in relation to cultural evolutionary theories of religion.

## Materials and Methods

### Grapheme Sample

The data used in this study derive from Classic Maya inscriptions recorded in the Maya Hieroglyphic Database [43]. This comprehensive compilation and concordance of Maya texts currently contains 65,269 records from inscribed architectural elements (i.e., stelae, stairways, panels, and murals), portable vessels such as painted pottery, and small objects made of jadeite, shell, bone, and wood. Each record in the database represents a glyph block, which is a graphically defined unit of text composed of one or more graphemes, each of which is individually coded. A complete catalog of all graphemes used in the Classic Maya inscriptions has been published by Macri and Looper [44].

**Figure 3:**
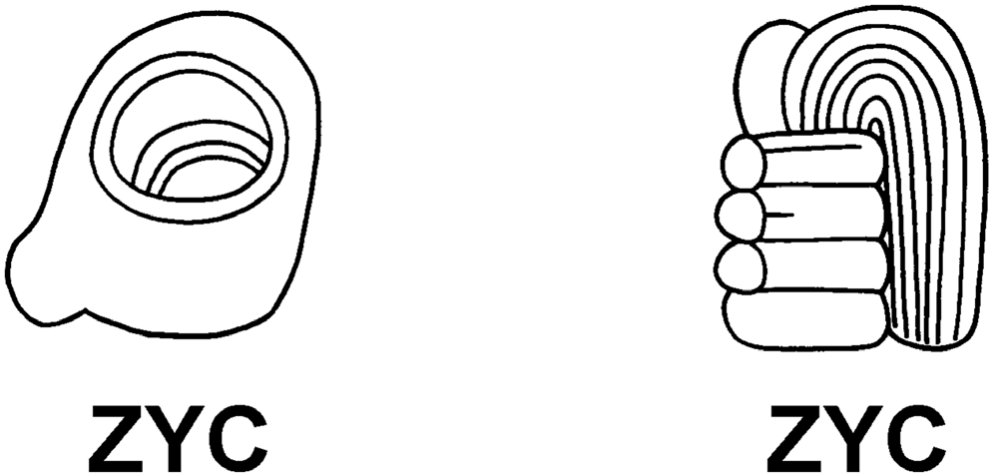
Detail of ZYC graphemes showing variants of the *ch’ahb’* bloodletting glyph. Drawing by Matthew G. Looper [44].

In this study, hieroglyphic evidence for bloodletting is based on a grapheme labeled ZYC in the MHD [44]. This logographic sign depicts a perforator or obsidian bloodletter, and occurs in two forms (Figure 3). Front and side views of the sign represent a three-dimensional object with a blade-like protuberance. In this form, the sign is usually depicted with an infixed circle and diagonal line which gives the appearance of an object that is polished or shiny, in this case probably obsidian. A less common variant of ZYC is the depiction of a bundled or wrapped bloodletter with feathered plumes attached to the instrument. Iconographically, both forms of the sign appear in scenes of ritual sacrifice and alongside other objects associated with bloodletting including stingray spines and strips of blood-stained paper (see Figure 2) [8], [20].

The ZYC grapheme is recorded a total of 89 times in the MHD. Of these, two examples are preceded by the phonetic complement **ch’a** (2G2) while **b’a** (XE1) appears beneath the perforator sign in 57 cases. For this reason, the current decipherment of the logograph is *ch’ahb’* [45]. Based on its lack of verbal affixation and its position within clauses in the hieroglyphic inscriptions, *ch’ahb’* is considered to be a noun. Words in the Greater Cholan and Yukatekan Mayan languages indicated by the **ch’a-** and **b’a-** phonetic complements on the perforator glyph ZYC are listed in Table 1. In Ch’olan the word *ch’ahb’* means ‘penance,’ which is a term not inappropriate to the notion of autosacrifice. This word may be related to the Yukatekan word *ch’ab’,* ‘dripper/dropper’, which is a derivation from the verb root *ch’a’,* meaning ‘to drip’. The noun *ch’ab’* is formed by adding the instrumental suffix -V*b’* to the verb root. Examples of the root *ch’aj* can be found in all four Yukatekan languages: Yukatek, Itzaj, Mopan, and Lakantun (see Table 1). The Yukatekan instrumental form in Yukatek, Itzaj, and Lakantun may be more precisely associated with sacrificial acts than the generalized Ch’olan term implies.

**Table 1:** Mayan words and their definitions associated with the transcription of the ZYC grapheme *ch’ahb’* referring to bloodletting.

MAYA WORD	TRANSLATION	LANGUAGE	DICTIONARY
***ch'aj*** ** forms related to ‘fast’**			
ch'ahb'	fast (ayuno)	proto-Cholan	[72] Kaufman (2003)
ch'ab'	prayer	Tzeltal	[73] Berlin and Kaufman (1990∶6)
ch'ab'	fast	Colonial Tzotzil	[74] Laughlin (1988∶194)
***ch’aj*** ** forms related to ‘drip’**			
ch'ajb'a'	lavar	Mopan	[75] Hofling (2011∶163)
ch'aj	gotear	Itzaj	[76] Hofling (1997∶211)
ch'ajb'äl	gotearlo	Itzaj	[76] Hofling (1997∶211)
ch'ajb'il	goteado	Itzaj	[54] Hofling (1997∶211)
ch'aj	goto, caida de gotas	Itzaj	[54] Hofling (1997∶211)
ch'ah	gota de agua, orina, o otro licor; gota decualquier licor o resina de árbol	Yukatek	[77] Barrera Basquez et al. (1997∶121)
ch'ah	drip	Yukatek	[78] Bricker (1998∶78)
ch'áah	drop	Yukatek	[78] Bricker (1998∶78)
ach'uhr ch'ah e ha'	water drips	Chorti'	[79] Wisdom (1950)
ch'ab'j	chapotear en el agua [splash in water]	Chuj	[80] Felipe Diego (1998∶55)
**Instrumental forms based on ** ***ch'aj***			
sh ch'ahab'	instr. eyedropper	Yukatek	[56] Bricker (1998∶78)
ch'ajab'aar	dropper	Lakantun	Hofling (personal communication)
***ch'oj*** **forms related to ‘bloodletting’**			
ch'oj	to chisel, drip, engrave, perforate, pierce	Colonial Tzotzil	[52] Laughlin (1988∶199)
ch'ojobil te'	chisel [perforator; piercer]	Colonial Tzotzil	[52] Laughlin (1988∶19)

**Figure 4:**
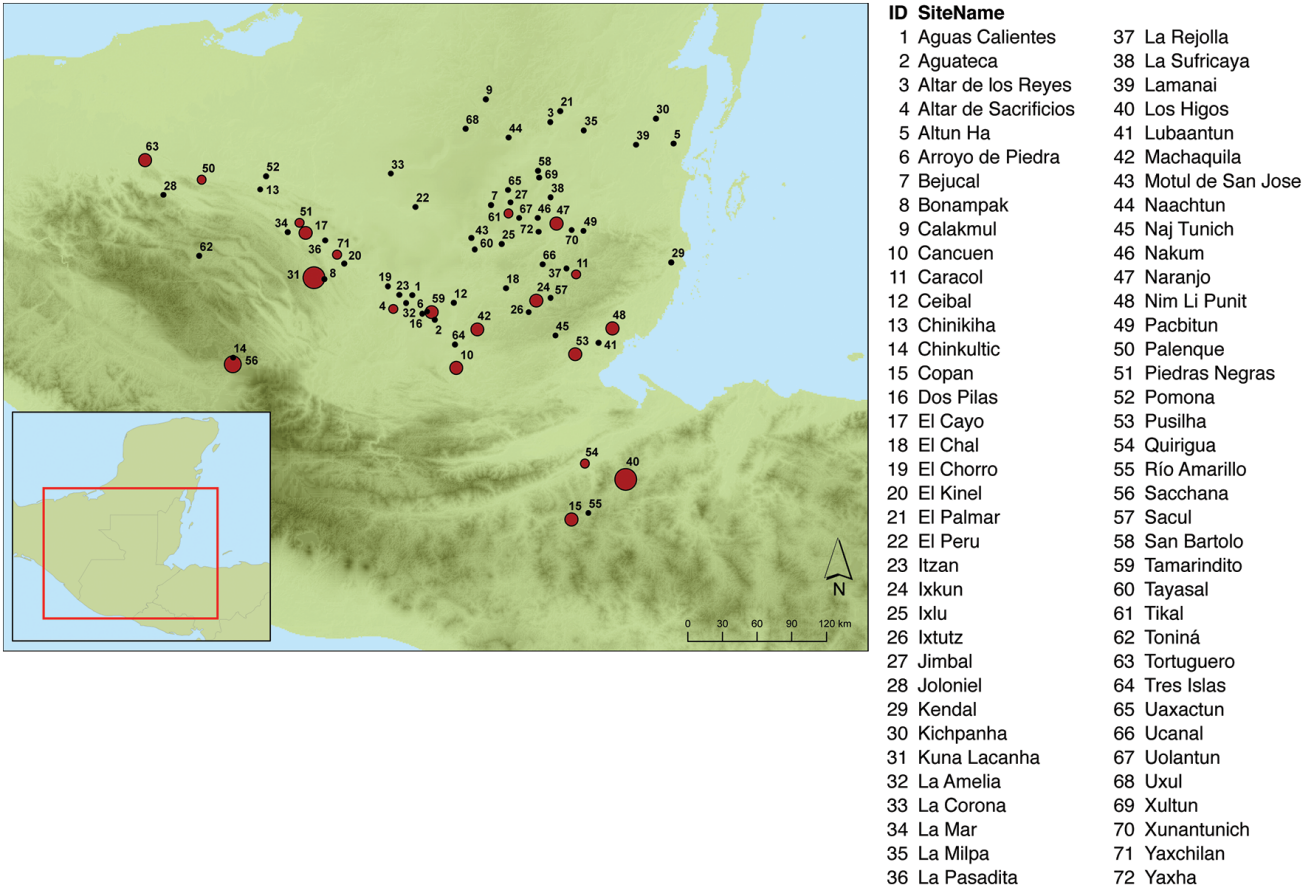
Location and name of southern Maya lowland sites included in the study. Red dot indicates sites that record the *ch’ahb’* bloodletting glyph and black dot indicates sites where no reference to *ch’ahb’* bloodletting has been found. Size of red dot refers to the relative abundance of dated monuments that record bloodletting rituals.

References to bloodletting were identified from 2480 non-portable architectural features included in the MHD. To ensure spatial and temporal reliability, we restricted the sample to 72 sites in the southern Maya region (Figure 4) where hieroglyphic monuments were produced during the Classic period (08.12.0.0.0 to 10.3.0.0.0 in the Long Count calendar system; 278–889 CE using the Goodman-Martinéz-Thompson correlation coefficient). From these 909 attributed monuments we identified 89 examples of the *ch’ahb’* glyph, 69 of which appeared to be directly associated with bloodletting (Table S1). For the latter 69 examples, we collected additional historical and social network data describing the associated contexts in which bloodletting statements were recorded (Tables S2 and S3). Geographic coordinates for all of the sites were obtained from the Electronic Atlas of Ancient Maya Sites [46].

### Spatial Analysis

We evaluated the spatial distribution of bloodletting records using a set of statistics known as local indicators of spatial association (LISA) [47]. In contrast to global measures of spatial autocorrelation, LISA statistics identify where significant spatial patterning occurs by quantitatively characterizing patterns of spatial dependence at multiple scales. In particular, we used two complementary statistics to identify local spatial concentrations of bloodletting records.

The first statistic, local Moran’s *I_i_*, is an index of similarity between sites with target values like those displayed at adjacent locales [47]. Positive *I_i_* scores indicate spatial clustering of similar values (i.e., either relatively high or low frequencies of *ch’ahb’*) while negative *I_i_* scores represent clustering of dissimilar values (e.g., a site with relatively high frequency of bloodletting records surrounded by sites with no *ch’ahb’* inscriptions).

The second statistic, Getis-Ord G_i_*, identifies spatial clusters of either high or low values by computing z-scores [48]. For statistically significant positive z-scores, large scores indicate more intense clustering of high values. For statistically significant negative z-scores, small scores indicate intense clustering of low values.

### Social Networks

We collected social network data describing specific sociopolitical relationships between sites to evaluate the related contexts and pathways in which bloodletting statements were recorded and spread. Ties between sites are based upon the appearance of foreign emblem glyphs and toponyms in hieroglyphic statements describing specific types of sociopolitical relations between Maya polities. Maya scholars generally agree that emblem glyphs refer to large political units, whether defined geographically or along kinship lines [49]. Emblem glyphs share a common structure in that they are composed of three elements: two constants, the glyphs *k’uhul* ‘divine’ or ‘holy’ and *ajaw* ‘lord’; and a third variable main sign of the particular kingdom or polity. Emblem glyphs usually follow the personal name of royal individuals mentioned in the texts and thus are generally understood as royal titles [50]. However, the main sign can also appear in other contexts where it serves as a toponym or place name [51]. In some cases, these signs appear at sites other than the one to which they refer. It is generally agreed that in such instances, these “foreign” emblem glyphs indicate an interaction or relationship between the individual agent or local site and the one referenced in the text [52]–[54]. We use the presence of these local and non-local place names in the MHD to collect data on specific network ties.

Network ties were assigned relational values based upon the semantic contexts of non-local place names and were previously classified by Munson and Macri [55], [56]. Ties fall into one of seven defined themes: antagonistic, diplomatic, dynastic, subordinate, kin, neutral and unknown statements (see Tables S2). There are too few ties in these networks to adequately evaluate their individual contribution to the spread of bloodletting, so all relations are weighted equally and a binary matrix (i.e., 1 if there is at least one tie among a pair of sites and 0 otherwise) was computed from the condensed relational data table. This dichotomized network is composed of 113 unique ties among 48 sites; 24 sites do not record a foreign place-name glyph and are identified as isolates in the network. An attribute table recording the presence and absence of the *ch’ahb’* logograph was used to partition the nodes in the visualized network. These matrices were used in a QAP correlation procedure to assess the similarity between them and test for homophily [57], [58]. Similarity was measured using a simple matching coefficient, which calculates the proportion of shared attributes in two dichotomous matrices *i* and *j*, and is defined by the equation:
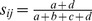
(1)where *a* is the number of dyads when *i* and *j* are both 1, *b* is the number of dyads where *i* is 1 and *j* is 0, *c* is the number of dyads where *i* is 0 and *j* is 1, and *d* is the number of dyads where both *i* and *j* are 0. The data were randomly permutated 10,000 times using the network and partition sizes to generate significance tests.

### Generalized Linear Mixed Model

We used a generalized linear mixed model (GLMM) to investigate the effects of several predictor variables on the frequency of *ch’ahb’* bloodletting statements. Specifically, we are interested in the historical contexts in which bloodletting rituals were recorded, so we included the aforementioned sociopolitical themes as fixed effects in the model and standardized these variables to account for their different ranges. Given the characteristics of this dataset, an ordinary linear regression would be an inappropriate model choice because assumptions about the Normal error distribution and the relation between the dependent and independent variables are violated for dichotomous or count data. These assumptions can be relaxed using Generalized Linear Models (GLMs), which include the ordinary linear regression model as a special case. GLMs, such as logistic regression or Poisson regression, differ from ordinary linear regression models in that they can incorporate various error distributions as well as nonlinear relationships among the response and the explanatory variables using a link function. Another assumption of GLMs pertains to the independence of observations. In this case, multiple observations of *ch’ahb’* statements per time period and site introduces correlation among the observations. To account for this potential correlation problem, we added random coefficients to the model corresponding to time and spatial location. The resulting model is thus a Generalized Linear Mixed Model (GLMM).

We now consider the specification of the model. When dealing with count data, two error distributions are plausible: the Poisson and the Negative Binomial distribution. The latter is particularly suitable for our data. In fact, a χ^2^ goodness of fit test rejected the null hypothesis of the Poisson distribution (χ^2^ = 45.6, df. = 3, p-value<0.01). This is due to overdispersion in the dataset, i.e., the variance of the data is greater than the mean (Likelihood ratio test for overdispersion LR = 11.9, sig. <0.01, parameter for overdispersion = 1.506). Consequently, we used a Negative Binomial mixed model. Because we are interested in the rate of *ch’ahb’* bloodletting statements with respect to the number of dated monuments at each site, we also introduced an offset variable in the linear predictor. Consequently, denoting fixed effects by *x*, random effects by *z*, and the variable related to the number of dated monuments by *m_i_*, the linear predictor 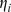 of the model can be specified by the following equation:
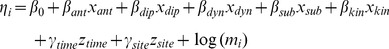
(2)where 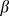 and 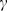 are the parameters related to the fixed and random effects, respectively. A log-link function is used to relate the expected value of the response variable to the linear predictor.

## Results

**Figure 5:**
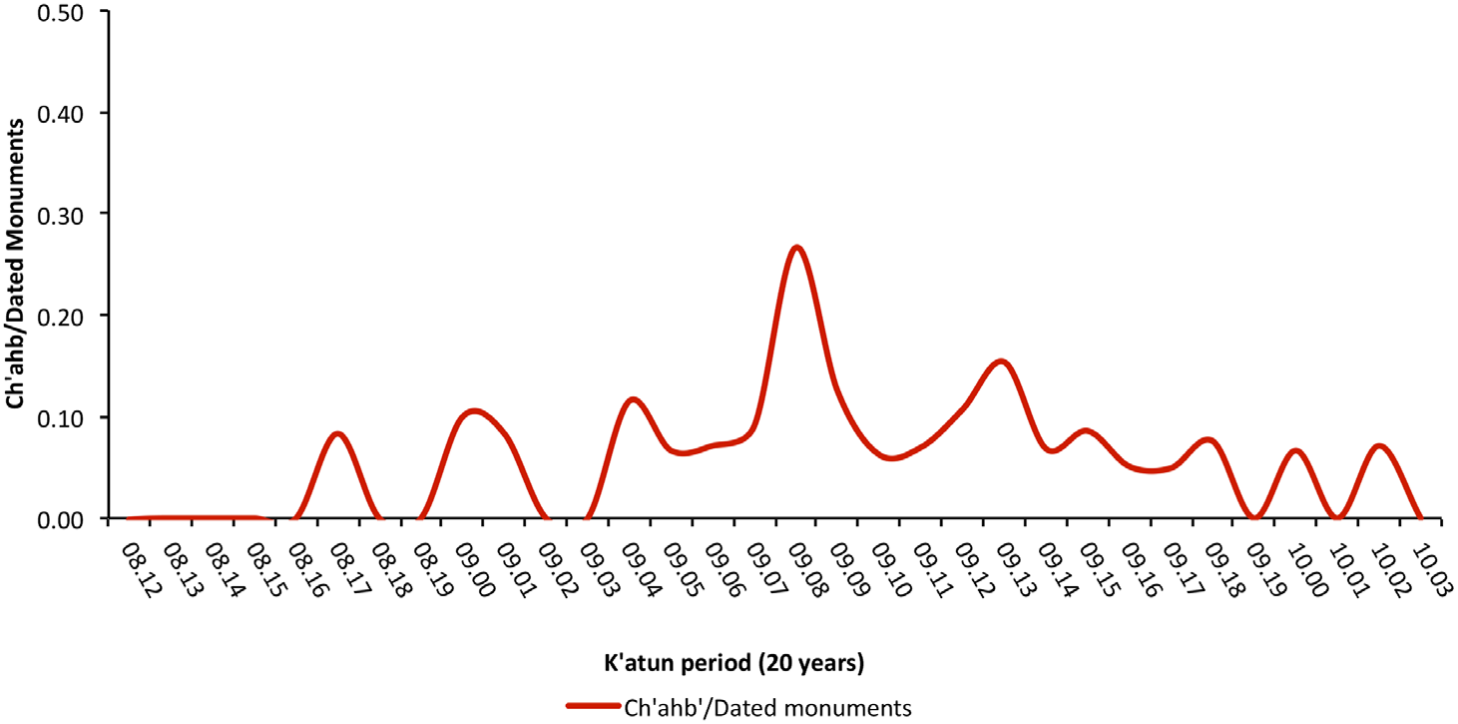
Relative frequency of dated monuments with *ch’ahb’* bloodletting glyph in each 20-year *k’atun* period. References to bloodletting rituals exceed 15% during two *k’atun* periods, 9.8.0.0.0 (593–613 CE) and 9.13.0.0.0 (692–712 CE).

The relative frequency of bloodletting statements recorded on dated monuments varies markedly through space and time (Figures 4 and 5). The distribution of southern Maya sites with recorded bloodletting is shown in Figure 4. Clearly, *ch’ahb’* bloodletting statements were not recorded with equal frequency at all site locations. Despite numerous sites with dated monuments, references to bloodletting are noticeably sparse in the central Petén region except at Tikal and Naranjo. Bloodletting statements are more common at sites in the southeastern region and along the Usumacinta River. In particular, it is noteworthy that 42% of all documented bloodletting rituals were recorded at just two sites: Copan (n = 18) and Yaxchilan (n = 11).

**Figure 6:**
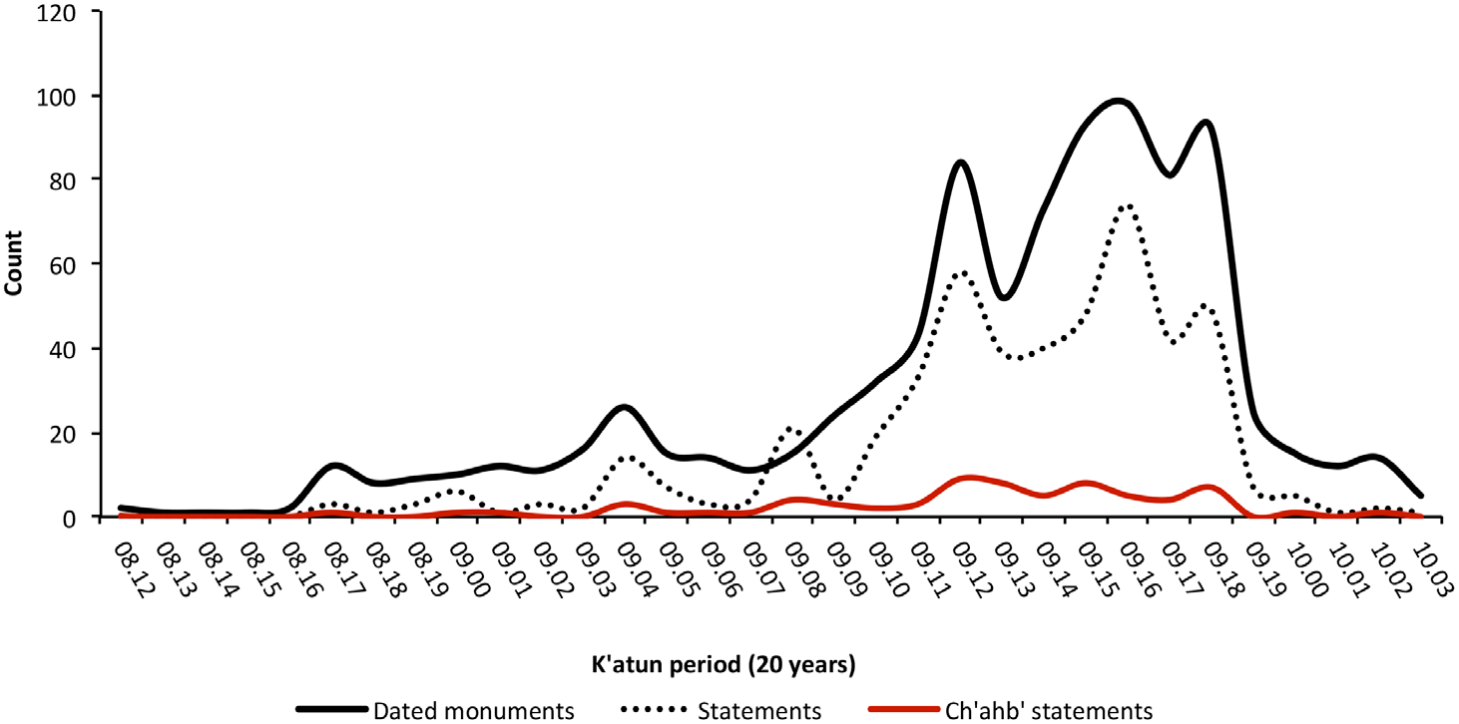
Temporal distribution of dated monuments, political statements, and *ch’ahb’* bloodletting statements included in the study. X-axis is divided into 20-year increments known as *k’atun* periods in the Maya Long Count calendar system. Note the overall low frequency of *ch’ahb’* bloodletting statements in comparison to the number of dated monuments during each *k’atun* period.

For much of the Classic period, *ch’ahb’* bloodletting statements occur at a low rate, but there are two phases when bloodletting references occur on more than 15% of all dated inscriptions. The chronological distribution of dated monuments sampled by the MHD is shown in Figure 6. Peak production in writing and construction of hieroglyphic monuments occurs during a 200-year period corresponding to the apogee of late Classic Maya civilization (ca. 600–800 CE). Variation in the total number of political statements recorded on these monuments follows this pattern [55]. However, variation in the number of *ch’ahb’* bloodletting statements does not. This suggests that changes in the frequency of bloodletting records are not simply a function of increased monument production through time. This inference is supported by the exclusion of the time variable as a random effect in the model described below.

**Figure 7:**
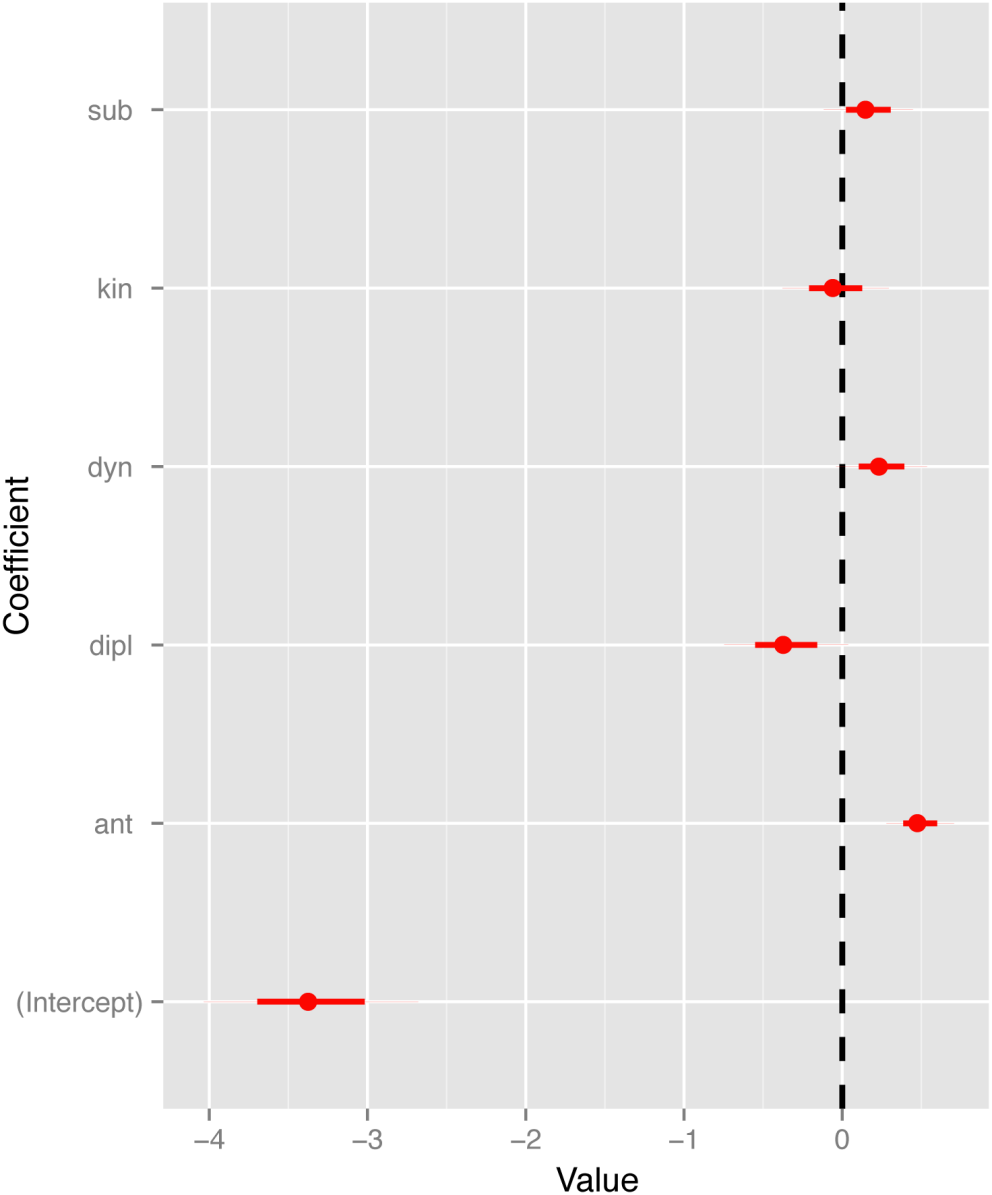
Estimates for the negative binomial mixed model effects including the confidence intervals.

To investigate the effects of these and other latent variables, we used a Negative Binomial mixed model to identify the sociopolitical contexts most closely associated with records of ritual bloodletting. We employed the same set of network themes used to construct the social network as predictor variables in this model. In light of recent research on the function of costly religious rituals and their role in cultural evolution [33], [59] we are especially interested in the relationship between bloodletting and statements concerning antagonistic and dynastic affairs. A GLMM specified by a log link function and a Negative Binomial error distribution was used to estimate the main effects of each sociopolitical theme (*x*) on the total number of *ch’ahb’* bloodletting statements (*y*). In order to select the best model, we used a forward procedure (i.e., starting with the null model, then adding the theme variables and finally the random effects) and compared their resulting AIC values using a likelihood ratio test. Table 2 shows that the model with the lowest AIC value includes all of the sociopolitical variables as well as the random effect related to geographic location. The estimates for the model are reported in Table 3 and visualized in Figure 7 together with their confidence intervals.

**Table 2:** Negative binomial mixed models including the fixed effects (*β*) and random effects (*γ*) with corresponding AIC values.

Model	Model specification	AIC
1	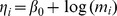	291.2
2	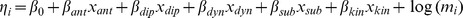	279.7**
3	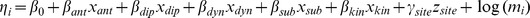	272.1*
4	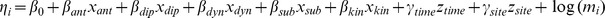	274.1

Sig. **0.001, *0.01.

The offset variable (*m_i_*) accounts for the number of dated monuments on which the observations were made. AIC values were tested using a Likelihood ratio test.

**Table 3:** Results of the Generalized Linear Mixed Model.

	β	s.e.(β)	Exp.(β)	CI Exp.(β) 2.5%	CI Exp.(β) 97.5%
**Intercept**	–3.37**	0.34	0.034	0.018	0.07
**Antagonistic**	0.47**	0.11	1.61	1.30	1.98
**Diplomatic**	–0.37*	0.20	0.69	0.47	1.01
**Dynastic**	0.23	0.14	1.26	0.95	1.67
**Kinship**	–0.06	0.17	0.94	0.68	1.31
**Subordinate**	015	0.14	1.16	0.88	1.53

Random coefficient (γ_0_) Variance 0.6745, s.d. 0.8213.

Sig. **<0.01, *<0.1.

Estimate values greater than 1 indicate positive correlation between the sociopolitical variable and the dependent variable, *ch’ahb’*. Values less than 1 indicate a negative relationship. Model fitting indicates that antagonistic statements are the only significant variable associated with bloodletting records.

Results indicate that antagonistic statements best predict the occurrence of *ch’ahb’* bloodletting records (Table 2). A likelihood ratio test shows that only the coefficient related to the number of antagonistic statements is significantly different from 0. The parameter related to diplomatic statements is only weakly significant at the 0.1 level. It is interesting to observe that while antagonistic ties have a positive effect, diplomatic ties have a negative effect on the rate of *ch’ahb’* bloodletting statements. Concerning random effects, the model estimates show that there is no variation in the rate of *ch’ahb’* bloodletting statements with respect to time, while there is variation with respect to geographic location. We interpret this to mean that there are sites which show a higher rate of *ch’ahb’* bloodletting statements compared to others as can be observed in Figure 4.

To investigate the processes responsible for the spread of bloodletting rituals we first tested a set of distance decay models for the spread of cultural traits using local spatial autocorrelation statistics. Inclusion of a random effect for spatial location in the model discussed above suggests that geography may have played a role in the diffusion of bloodletting. Previous studies indicate that Maya polities engaged in sociopolitical interactions at a range of spatial scales [56], [60], so we examined a series of lagged distances at 25-km intervals to determine whether bloodletting rituals exhibit similar spatial heterogeneity. For this we computed two measures of local spatial autocorrelation, Moran’s *I*_i_ and Getis-Ord G_i_*. The former is an index of similarity used to compare attribute values between neighboring sites [47]. In the present study, we are interested in sites with large positive *I*_i_ scores, which indicate positive local spatial autocorrelation between a target site and neighboring sites that display similar frequencies of bloodletting references. Getis-Ord G_i_* is a complementary measure that is used to identify spatial clusters of sites with relatively high and low attribute values [48]. We employed Getis-Ord G_i_* as a relative measure of the total number of *ch’ahb’* bloodletting statements within a target site’s neighborhood and used it to identify local regions of autocorrelation containing relatively high or low quantities of bloodletting references. In both analyses, we used the relative frequency of *ch’ahb’* statements as a function of the total number of dated monuments at each site to ensure comparability of attribute values across the sample.

**Figure 8:**
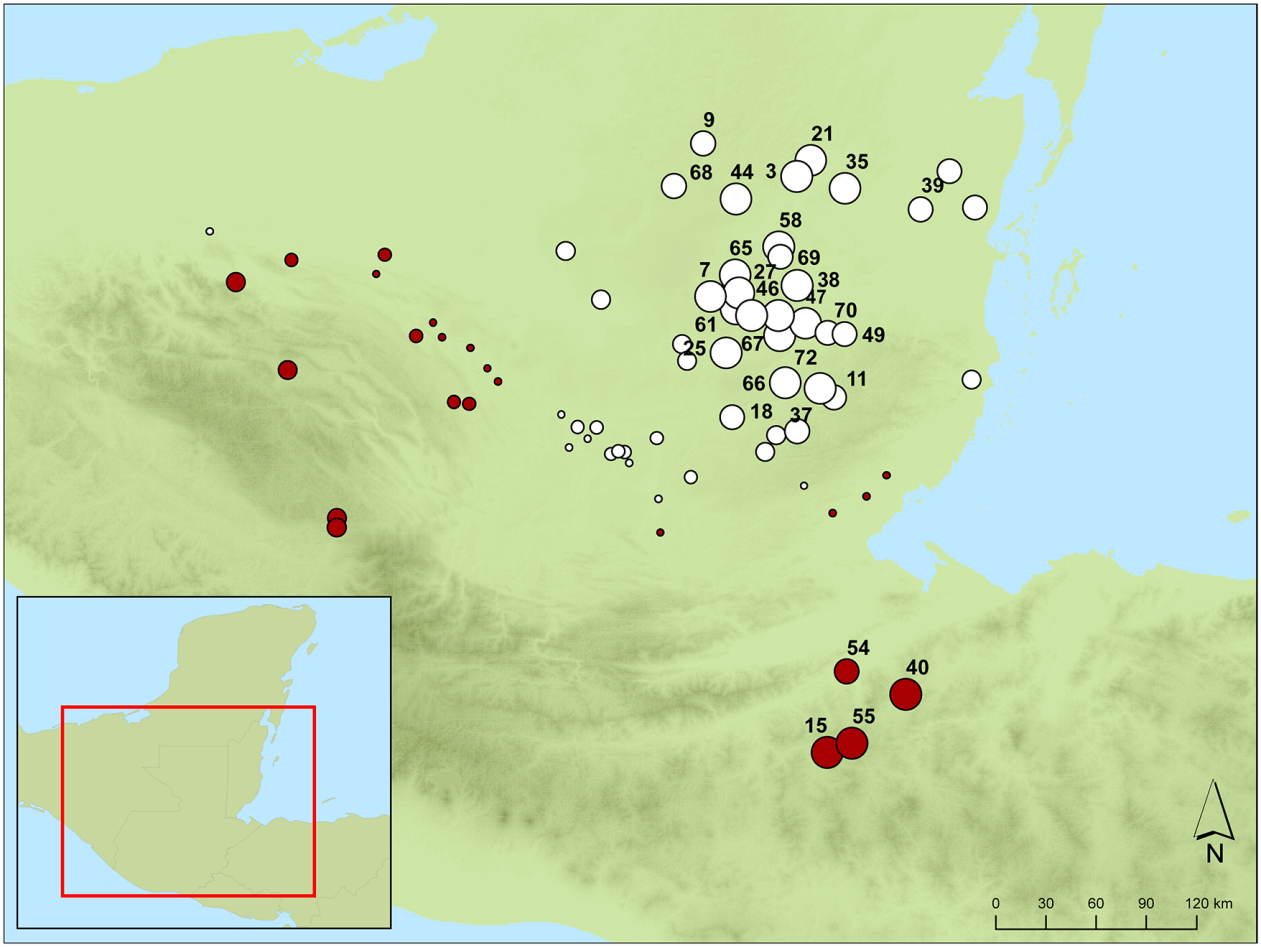
Bubble graph of standardized G_i_* scores at a lag distance of 150 kilometers. Bubble area is proportional to |G_i_*|. Red bubbles represent positive G_i_* scores, where *ch’ahb’* bloodletting rituals are recorded. White bubbles represent negative G_i_* scores, where there are no bloodletting records. Sites labeled with ID number have significant G_i_* scores and correspond to the site names and IDs in Figure 4.

Although previous studies indicate that Classic Maya sites within 75 km of each other are most likely to compete and hold similar social positions [39], [56], sites that record the *ch’ahb’* bloodletting glyph do not display significant *I*_i_ scores at this distance (Table S4). Doubling the spatial lag to remove sampling effects due to small neighborhood sizes does not greatly alter this pattern. The results indicate that *ch’ahb’* bloodletting statements do not display spatial dependency at any geographic scale. However, a plot of standardized G_i_* scores reveals significant regional clusters of sites with low and high concentrations of *ch’ahb’* statements at a spatial lag of 150 km (Figure 8). Negative scores in the central Petén area define a large region where bloodletting rituals were rarely recorded. Significant positive scores in the southeastern area represent a small concentration of sites heavily invested in recording *ch’ahb’* statements. Combined, these results suggest that the spread of bloodletting rituals was not simply a consequence of Classic Maya royals copying their neighbors.

To determine what other mechanisms may be responsible for the spread of Classic Maya bloodletting, we investigated the impact of social ties on the occurrence of *ch’ahb’* statements. Homophily refers to the general principle that similarity promotes connection, which has profound implications for the transmission of cultural information in social networks [61]. It means that any attribute that depends on networks to spread will tend to be localized in social space and will result in a predictable network structure [62]. In a homophilous network, connections among sites that share some attribute ought to occur at a higher rate than among sites that do not share the attribute.

**Figure 9:**
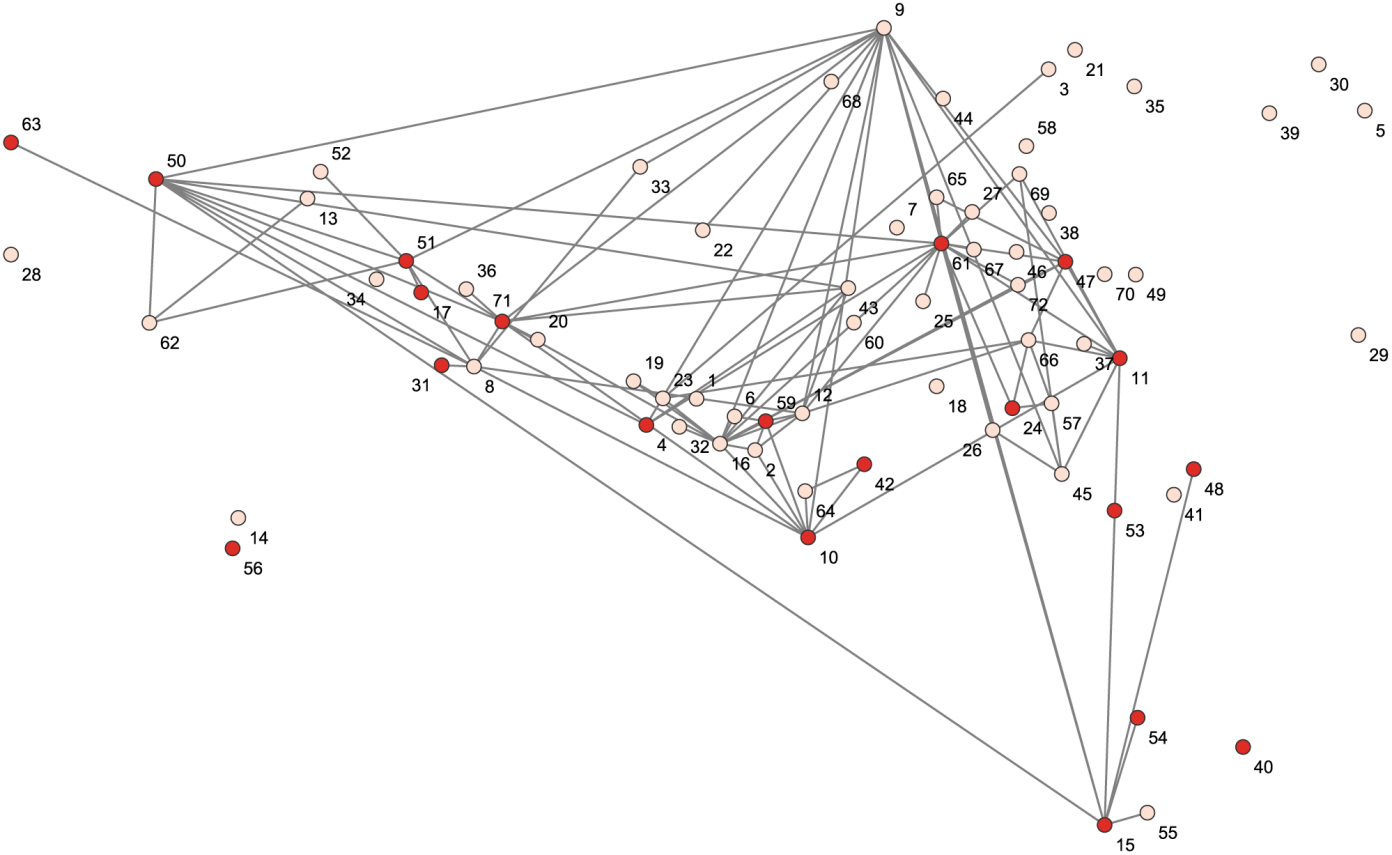
Network graph showing the sociopolitical ties between sites partitioned by the presence of *ch’ahb’* bloodletting statements. Sites labeled in red indicate the presence of *ch’ahb’* (light pink indicates absence). The nodes are plotted using their geographic coordinates. Site IDs correspond to the names listed in Figure 4.

For this study we are interested in whether sites that record bloodletting are more likely to also share a dyadic tie. Dyadic ties are based upon the appearance of place-name glyphs in hieroglyphic statements that describe social and political relations between Maya polities [55]. Figure 9 shows a graph of the network partitioned by the presence of *ch’ahb’* bloodletting statements. Results of the QAP correlation indicate a strong and positive relationship between network ties and bloodletting (*s_ij_* = 0.909, p = 0.001). Sites that record bloodletting statements thus exhibit a strong tendency to have ties with like others. In addition, pairs of sites where *ch’ahb’* statements are not present are less likely to share a network tie. These findings support the existence of homophily in this dataset, and suggest that social and political ties among Classic Maya centers contributed to the transmission of these rituals. The existence of homophily, however, does not allow us to identify causality or differentiate between selection (where ties are created among sites that record bloodletting) and influence mechanisms (in which linked sites adopt bloodletting) in the spread of ritual bloodletting. Indeed, disambiguating these causal processes remains an extant challenge for social network analysis [63].

## Discussion

Ritual bloodletting is widely regarded as a central feature in the evolution of Classic Maya society [1]. This ancient ritual practice is also cited as a key example in more general discussions about the role of costly signals in the cultural evolution of religion [9], [10]. To assess these generalizations, we need to understand the specific mechanisms and contexts in which bloodletting occurred. This study provides the first systematic account of bloodletting rituals drawn from the Classic Maya hieroglyphic record, and demonstrates how important insights on the evolution of religious rituals can be gained from quantitative studies of the spatial and temporal occurrences of this glyph.

Previous descriptions of Classic Maya bloodletting portray this ritual as a nearly ubiquitous act of self-sacrifice performed by Maya rulers. These ritual practices are considered to be essential–if not costly–rites of passage that signaled specific qualities of young royals as well as their commitments to their new role in Classic Maya society [21], [29], [30]. The results of our study cast doubt on the widespread distribution of these dynastic rituals and suggest that bloodletting, as a costly signal, was much more restricted in Classic Maya society. In particular, the results of our model identify geographic location and antagonism as primary predictors for the occurrence of inscribed *ch’ahb’* glyphs on dated monuments. As discussed above, records of bloodletting are predominantly found at a small number of sites along the Usumacinta River and in the southeastern region of the Maya lowlands. Although the actual *practice* of bloodletting may have been more common than the inscriptions account for, this study captures only those unambiguous instances of recorded bloodletting rituals. The tradition of recording these costly displays underscores one proposed function of Maya hieroglyphic monuments as important transmitters of cultural information. We argue that the documentation of these rituals on public monuments can be interpreted as conspicuous messages about specific dynastic lineages. However, we did not find a universal relationship between *ch’ahb’* glyphs and dynastic statements referring to royal accession. Instead, we identified antagonistic statements about warfare and conflict to be the primary contexts in which bloodletting is mentioned. This finding is compatible with earlier interpretations of Classic Maya bloodletting that associate warfare and the capture of prisoners with autosacrifice [17]. However, Demarest suggests that such related practices formed part of a larger set of dynastic rituals expected of proper Maya rulers [1]. Thus, we must also consider the possibility that antagonistic actions such as taking war captives represent another credible sign of a ruler’s power and dominance within Classic Maya society.

An alternative explanation for the spatial distribution of *ch’ahb’* glyphs and their relationship with antagonistic statements relies explicitly on hypotheses derived from signaling theory. The costly signaling theory of religion predicts that collective actions such as warfare pose potential free-rider problems such that reliable signals promoting solidarity and within-group cooperation should be highly favored [4]. Based on these ideas, Sosis and colleagues propose several hypotheses to explain cross-cultural differences in costly male rites including various types of scarification, piercing, and genital mutilation [41]. In particular, they hypothesize a positive link between warfare frequency and the costliness of male rites. Sosis et al. [41] posit a further relationship between specific types of warfare and the relative cost of these rituals: societies that engage in external warfare (i.e., conflicts with those outside their group) will require their males to bear more permanent marks than societies with only internal warfare (i.e., conflict that threatens the lives of individuals within the cultural group). Our model supports the first part of this hypothesis, but does not currently meet the more specific conditions of the second clause. Although we find a positive relationship between bloodletting and antagonistic statements, these declarations of war are generally directed towards rival polities and competing rulers within Maya society. Previous research on Maya warfare indicates this was an internal and personal affair–fought between ethnically and culturally similar antagonists who abided by mutually understood conventions [64]. According to the costly ritual-warfare hypothesis, we would expect communities that engage in frequent internal warfare to resist rituals that result in permanent marks–such as bloodletting–which can signal group identity, due to the mobility of individuals across kin groups and consequent shifting of alliances [41]. Our results suggest a different situation: rival rulers appear to have proudly displayed the markings of bloodletting despite crosscutting network ties. Interestingly though, bloodletting records are predominantly found in two peripheral zones of the Maya lowlands where contact and conflict with non-Maya groups may have been common. If so, it is possible that bloodletting rituals and the documentation of these displays represented a sign of group membership in culturally diverse environments. However, this hypothesis will have to await future evaluation. Another point that raises doubt about the costly ritual-warfare hypothesis for Classic Maya bloodletting relates to the gender of ritual practitioners. The study conducted by Sosis and colleagues was limited to rituals performed exclusively by males [41]. At least a subset of bloodletting statements seem to refer to male initiation rites (*yax ch’ahb’* ‘first bloodletting’) [29], although we cannot be certain that all *ch’ahb’* statements refer only to male initiation rites. This study does not identify whether males or females were the main subjects of bloodletting statements, but we know that this ritual was not exclusive to male practitioners (see Figure 1). Thus, we must consider the diverse roles of ritual practitioners according to gender, age, and other variables in future studies of Classic Maya bloodletting.

Beyond basic contextual clues about the conditions surrounding Classic Maya bloodletting, results of this study also allow us to evaluate possible pathways for the spread of this ritual practice. While the spatial distribution of bloodletting records is noteworthy, this pattern is not consistent with mechanisms of spatial diffusion. Instead, by measuring the social distance between sites we found that network ties are strongly correlated with bloodletting statements. In other words, sites where bloodletting was recorded are highly connected. Thus, bloodletting rituals seem to have spread through social network ties rather than a process of spatial diffusion. However, a basic pattern of homophily is not enough to distinguish whether selection processes or influence processes are primarily responsible for the transmission of cultural traits [63]. Given the current dataset, a process of selection would create network ties among sites that share the cultural attribute of recording *ch’ahb’* glyphs, whereas a process of social contagion (also called influence) works in the opposite direction with sites adopting the cultural practice based on an existing network tie. There are few models capable of dealing with the problem of separating these transmission processes [65], [66]. Moreover, these models are quite complex, requiring a specific set of data in which time plays a central role. Although the dataset used in this study is time-sensitive, it does not currently meet the requirements specified by these dynamic models. However, given the relatively low occurrence of *ch’ahb’* glyphs in the overall corpus of Maya hieroglyphic texts, it is unlikely that this single variable drove the formation of network ties. Rather, the spread of bloodletting appears to result from a form of social contagion between connected polities. Network ties between sites thus identify potential transmission pathways of bloodletting rituals that included kinship lines, marriage exchanges, subjugation, and political alliances between royal courts.

Religious rituals that are painful or highly stressful are often considered to be costly signs of commitment that promoted cooperation, thereby contributing to the evolution of large-scale societies over the past 10,000 years [67]–[70]. In general though these theories do not address how such extravagant displays varied over time and between different social groups in the past–historical information that is essential for studying the evolution of religion. This study demonstrates the first attempt to quantitatively analyze patterns of Classic Maya bloodletting recorded in hieroglyphic texts. Results of the present study illustrate the historical dynamics of these ancient rituals and offer clues to the variable contexts and consequences of religious rituals beyond samples drawn from contemporary Western societies. Moreover, this study highlights the need for archaeologists and historians to participate in debates about the evolutionary origins and cognitive science of religion [71].

## Supporting Information

Table S1 **Records of bloodletting included in the study.**
(DOCX)

Table S2 **Classification schema for building the social network using relational statements employing toponyms and emblem glyphs.** The theme category represents seven different types of sociopolitical relationships identified and analyzed in this and previous studies [55], [56].
(DOCX)

Table S3 **Relative frequency of sociopolitical statements in MHD based on the number of dated monuments in each 20-year** ***k’atun*** **period.**
(DOCX)

Table S4 **Calculated** ***I_i_*** **and** G_i_* **scores at all 25 km spatial lags. Significant values indicated in bold.**
(DOCX)
