# Supplementary material for: Classic Maya Bloodletting and the Cultural Evolution of Religious Rituals: Quantifying Patterns of Variation in Hieroglyphic Texts
Source: PLoS One. 2014 Sep 25;9(9):e107982. doi: 10.1371/journal.pone.0107982 (PMC4177853; doi:10.1371/journal.pone.0107982)
Supplement: Table S1 — Records of bloodletting included in the study. (DOCX) [file pone.0107982.s001.docx]

**Table S1.** Records of bloodletting included in the study.

| **ID** | **Site Name** | **Monument Name** | **Long Count Date** | **Katun** |
| --- | --- | --- | --- | --- |
| 1 | Altar de Sacrificios | Stela 04 | 09.10.03.17.00 | 09.10 |
| 2 | Cancuen | Panel | 09.12.10.00.00 | 09.12 |
| 3 | Caracol | Stela 03 | 09.07.19.13.12 | 09.07 |
| 4 | Caracol | Stela 01 | 09.08.00.00.00 | 09.08 |
| 5 | Copan | Motmot Capstone | 09.00.00.00.00 | 09.00 |
| 6 | Copan | Papagayo Step | 09.01.10.00.00 | 09.01 |
| 7 | Copan | Altar A' | 09.04.99 | 09.04 |
| 8 | Copan | Altar L' | 09.04.99 | 09.04 |
| 9 | Copan | CPN 56/Stela 09 | 09.06.10.00.00 | 09.06 |
| 10 | Copan | CPN 29/Stela P | 09.09.10.00.00 | 09.09 |
| 11 | Copan | CPN 29/Stela P | 09.09.10.00.00 | 09.09 |
| 12 | Copan | CPN 54/Stela 07 | 09.09.00.00.00 | 09.09 |
| 13 | Copan | CPN 69/Stela 19 | 09.10.19.15.00 | 09.10 |
| 14 | Copan | CPN 56/Stela 10 | 09.11.00.00.00 | 09.11 |
| 15 | Copan | CPN 18/Stela I | 09.12.03.14.00 | 09.12 |
| 16 | Copan | CPN 52/Stela 06 | 09.12.10.00.00 | 09.12 |
| 17 | Copan | CPN 52/Stela 06 | 09.12.10.00.00 | 09.12 |
| 18 | Copan | CPN 87/Altar I' | 09.12.10.00.00 | 09.12 |
| 19 | Copan | CPN 11/Stela F | 09.14.10.00.00 | 09.14 |
| 20 | Copan | Temple 22, Hieroglyphic Step | 09.14.03.06.08 | 09.14 |
| 21 | Copan | CPN 34/Altar U | 09.17.09.02.12 | 09.17 |
| 22 | Copan | Structure 30 Altar | 09.17.00.00.00 | 09.17 |
| 23 | El Cayo | Lintel 01 | 09.16.12.02.06 | 09.16 |
| 24 | Ixkun | Stela 01 | 09.18.00.00.00 | 09.18 |
| 25 | Kuna Lacanha | Lintel 01 | 09.15.15.00.00 | 09.15 |
| 26 | Los Higos | Stela 01 | 09.17.10.07.00 | 09.17 |
| 27 | Machaquila | Stela 11 | 09.15.10.00.00 | 09.15 |
| 28 | Machaquila | Stela 06 | 10.00.05.00.00 | 10.00 |
| 29 | Naranjo | Stela 22 | 09.13.10.00.00 | 09.13 |
| 30 | Naranjo | Stela 23 | 09.13.18.04.18 | 09.13 |
| 31 | Naranjo | Stela 13 | 09.17.10.00.00 | 09.17 |
| 32 | Naranjo | Stela 08 | 09.18.10.00.00 | 09.18 |
| 33 | Naranjo | Stela 12 | 09.18.09.13.15 | 09.18 |
| 34 | Naranjo | Stela 35 | 09.18.09.00.13 | 09.18 |
| 35 | Nim Li Punit | Stela 15 | 09.14.10.00.00 | 09.14 |
| 36 | Nim Li Punit | Stela 21 | 09.18.00.00.00 | 09.18 |
| 37 | Palenque | Tablet of the Cross | 09.12.19.14.12 | 09.12 |
| 38 | Palenque | Tablet of the Sun | 09.12.19.14.12 | 09.12 |
| 39 | Palenque | Temple 19, Platform, south | 09.15.05.00.00 | 09.15 |
| 40 | Palenque | Tablet of the Foliated Cross | 09.12.18.05.19 | 09.12 |
| 41 | Palenque | Temple of the Cross | 09.12.10.00.00 | 09.12 |
| 42 | Palenque | Temple 21, Bench Edge | 09.13.17.09.00 | 09.13 |
| 43 | Piedras Negras | Lintel 12 | 09.04.03.10.01 | 09.04 |
| 44 | Piedras Negras | Stela 25 | 09.08.10.04.19 | 09.08 |
| 45 | Piedras Negras | Stela 25 | 09.08.10.04.19 | 09.08 |
| 46 | Pusilha | Stela D | 09.08.01.12.08 | 09.08 |
| 47 | Pusilha | Stela E | 09.11.00.00.00 | 09.11 |
| 48 | Pusilha | Stela E | 09.15.00.00.00 | 09.15 |
| 49 | Quirigua | Monument 10/Stela J | 09.16.05.00.00 | 09.16 |
| 50 | Sacchana | Stela 02 | 10.02.10.00.00 | 10.02 |
| 51 | Tamarindito | Hieroglyphic Stairway 03, Step 06 | 09.16.11.07.13 | 09.16 |
| 52 | Tikal | Stela 39 | 08.17.00.00.00 | 08.17 |
| 53 | Tikal | Stela 10 | 09.05.04.05.16 | 09.05 |
| 54 | Tikal | Temple I Lintel 03 | 09.13.03.09.18 | 09.13 |
| 55 | Tikal | Temple I Lintel 03 | 09.13.03.09.18 | 09.13 |
| 56 | Tikal | Column Altar 01 | 09.15.17.10.04 | 09.15 |
| 57 | Tikal | Temple IV Lintel 03 | 09.15.15.02.03 | 09.15 |
| 58 | Tortuguero | Monument 06 | 09.11.16.08.18 | 09.11 |
| 59 | Yaxchilan | Lintel 24 | 09.13.17.15.12 | 09.13 |
| 60 | Yaxchilan | Lintel 24 | 09.13.17.15.12 | 09.13 |
| 61 | Yaxchilan | Lintel 24 | 09.13.17.15.12 | 09.13 |
| 62 | Yaxchilan | Stela 18 | 09.14.17.15.12 | 09.14 |
| 63 | Yaxchilan | Stela 18 | 09.14.17.15.12 | 09.14 |
| 64 | Yaxchilan | Lintel 14 | 09.15.10.00.01 | 09.15 |
| 65 | Yaxchilan | Stela 35 | 09.15.10.00.01 | 09.15 |
| 66 | Yaxchilan | Stela 10 | 09.16.15.00.00? | 09.16 |
| 67 | Yaxchilan | Stela 11 | 09.16.01.00.00 | 09.16 |
| 68 | Yaxchilan | Lintel 10 | 09.18.17.12.06 | 09.18 |
| 69 | Yaxchilan | Lintel 10 | 09.18.17.12.06 | 09.18 |
