# Supplementary material for: Classic Maya Bloodletting and the Cultural Evolution of Religious Rituals: Quantifying Patterns of Variation in Hieroglyphic Texts
Source: PLoS One. 2014 Sep 25;9(9):e107982. doi: 10.1371/journal.pone.0107982 (PMC4177853; doi:10.1371/journal.pone.0107982)
Supplement: Table S2 — Classification schema for building the social network using relational statements employing toponyms and emblem glyphs. The theme category represents seven different types of sociopolitical relationships identified and analyzed in this and previous studies [55], [56]. (DOCX) [file pone.0107982.s002.docx]

**Table S2.** Classification schema for building the social network using relational statements employing toponyms and emblem glyphs. The theme category represents seven different types of sociopolitical relationships identified and analyzed in this and previous studies [[31](#_ENREF_31), [32](#_ENREF_32)].

| **THEME** | **TRANSLITERATION** | **CH’OLAN** | **ENGLISH GLOSS** |
| --- | --- | --- | --- |
| Antagonistic: hostile relationships involving subjugation | | |  |
|  | **u(-cha?)-chan(-nu)** | *uchan?* | ‘his guardian’ (+ captor emblem) |
|  | **u(-cha?)-chan(-nu)** | *uchan?* | ‘his guardian’ (+ captive emblem) |
|  | **ya-te-a** | *yate’ah?* | ‘prisoner’ |
|  | **pul-yi, pu-lu-yi** | *puluy* | ‘burned’ |
|  | **hub’?-yi** | *hub’uy?* | ‘fell’ |
|  | **chu-ka-ja** | *chukaj* | ‘was captured’ |
|  | **u-na-ka-wa** | *unak-wa* | ‘attacked’ |
|  | **u-ch’äk(-ja)** | *ch’äk* | ‘attacked, cut’ |
|  | **ya-la-ja** | *yalaj* | ‘threw him’ |
|  | **ja-tza-ja** | *jatzaj* | ‘was hit’ |
|  | **yäl** | *yäl* | ‘throw down’ |
|  | **el** | *el* | ‘burns’ |
|  | **tok' pakal** | *tok’ pakal* | ‘his flint his shield’ |
|  |  |  |  |
| Diplomatic: non-hostile, friendly relations often involving ritual practice | | | |
|  | **yi-ta-ji** | *yitaj* | ‘together with’ |
|  | **ye-te(-he)** | *yetel* | ‘by, with’ |
|  | **yi-chi-nal** | *yichnal* | ‘in his company’ |
|  | **pi-tzi-ja** | *pitzaj* | ‘played ball’ |
|  | **(yi-)il(-li)** | *il* | ‘witnessed’ |
|  | **chok ch'aj** | *chok ch'aj* | ‘scattering’ |
|  |  |  |  |
| Dynastic lineage: sovereign relations | | | |
|  | **chum-wa-ni ta ajaw-le** | *chumwan ti ajawlel* | ‘was seated in ajawship’ |
|  | **joy?-ja ti sa-ja-li** | *joyah? ti’ sajal* | ‘acceded as sajal’ |
|  | **ik hun?** | *ik’ hun* | ‘takes the headband’ |
|  | **ajawyan** | *ajawyan* | ‘accession’ |
|  | **joy?-ja ti ajaw-le** | *joyah? ti’ ajaw* | ‘acceded as ajaw’ |
|  |  |  |  |
| Kinship lineage: familial relations | |  |  |
|  | **ya-al(-la)** | *yal* | ‘her child’ |
|  | **u-nich?** | *unich?* | ‘his child’ |
|  | **ya-tz'i/xu?-na** | *??* | ‘his mother’ |
|  | **ya-ta-na** | *yatan* | ‘wife’ |
|  |  |  |  |
| Subordination: hierarchical relationship involving subjugation | | | |
|  | **ya-ha-wa, ya-ajaw** | *yajal* | ‘his ajaw’ |
|  | **sa-ja-la** | *sajal* | ‘noble title’ |
|  | **u-kab’-hi(-ya)** | *ukab’i/ukahi* | ‘under his authority’ |
|  |  |  |  |
| Neutral: impartial, equitable relationship often in the local instance when none of the other criteria are present | | | |
| Unknown: indiscernible due to erosion, decipherment, or lack of information | | | |
