# Supplementary material for: Classic Maya Bloodletting and the Cultural Evolution of Religious Rituals: Quantifying Patterns of Variation in Hieroglyphic Texts
Source: PLoS One. 2014 Sep 25;9(9):e107982. doi: 10.1371/journal.pone.0107982 (PMC4177853; doi:10.1371/journal.pone.0107982)
Supplement: Table S3 — Relative frequency of sociopolitical statements in MHD based on the number of dated monuments in each 20-year k’atun period. (DOCX) [file pone.0107982.s003.docx]

**Table S3.** Relative frequency of sociopolitical statements in MHD based on the number of dated monuments in each 20-year *k’atun* period.

| ***K'atun*** | **No. dated monuments** | **Antagonistic** | **Diplomatic** | **Dynastic** | **Kinship** | **Subordination** |
| --- | --- | --- | --- | --- | --- | --- |
| 08.12 | 2 | 0.000 | 0.000 | 0.000 | 0.000 | 0.000 |
| 08.13 | 1 | 0.000 | 0.000 | 0.000 | 0.000 | 0.000 |
| 08.14 | 1 | 0.000 | 0.000 | 0.000 | 0.000 | 0.000 |
| 08.15 | 1 | 0.000 | 0.000 | 0.000 | 0.000 | 0.000 |
| 08.16 | 2 | 0.000 | 0.000 | 0.000 | 0.000 | 0.000 |
| 08.17 | 12 | 0.000 | 0.000 | 0.167 | 0.000 | 0.000 |
| 08.18 | 8 | 0.000 | 0.000 | 0.125 | 0.000 | 0.000 |
| 08.19 | 9 | 0.000 | 0.111 | 0.222 | 0.111 | 0.111 |
| 09.00 | 10 | 0.000 | 0.100 | 0.400 | 0.000 | 0.100 |
| 09.01 | 12 | 0.000 | 0.000 | 0.000 | 0.000 | 0.000 |
| 09.02 | 11 | 0.000 | 0.000 | 0.273 | 0.000 | 0.000 |
| 09.03 | 16 | 0.000 | 0.000 | 0.125 | 0.000 | 0.000 |
| 09.04 | 26 | 0.000 | 0.038 | 0.385 | 0.000 | 0.000 |
| 09.05 | 15 | 0.000 | 0.200 | 0.200 | 0.000 | 0.333 |
| 09.06 | 14 | 0.071 | 0.000 | 0.071 | 0.143 | 0.000 |
| 09.07 | 11 | 0.091 | 0.091 | 0.091 | 0.000 | 0.091 |
| 09.08 | 15 | 0.133 | 0.133 | 0.867 | 0.000 | 0.067 |
| 09.09 | 24 | 0.000 | 0.000 | 0.042 | 0.000 | 0.083 |
| 09.10 | 32 | 0.188 | 0.125 | 0.219 | 0.063 | 0.219 |
| 09.11 | 43 | 0.070 | 0.047 | 0.581 | 0.047 | 0.047 |
| 09.12 | 84 | 0.119 | 0.012 | 0.452 | 0.083 | 0.071 |
| 09.13 | 52 | 0.154 | 0.019 | 0.423 | 0.096 | 0.038 |
| 09.14 | 73 | 0.137 | 0.041 | 0.301 | 0.164 | 0.151 |
| 09.15 | 93 | 0.269 | 0.043 | 0.118 | 0.054 | 0.108 |
| 09.16 | 98 | 0.357 | 0.010 | 0.337 | 0.041 | 0.041 |
| 09.17 | 81 | 0.148 | 0.025 | 0.296 | 0.062 | 0.148 |
| 09.18 | 92 | 0.261 | 0.011 | 0.185 | 0.043 | 0.217 |
| 09.19 | 25 | 0.080 | 0.040 | 0.160 | 0.000 | 0.080 |
| 10.00 | 15 | 0.200 | 0.067 | 0.000 | 0.000 | 0.000 |
| 10.01 | 12 | 0.000 | 0.083 | 0.000 | 0.000 | 0.083 |
| 10.02 | 14 | 0.000 | 0.071 | 0.000 | 0.000 | 0.000 |
| 10.03 | 5 | 0.000 | 0.200 | 0.000 | 0.000 | 0.000 |
