# Supplementary material for: Classic Maya Bloodletting and the Cultural Evolution of Religious Rituals: Quantifying Patterns of Variation in Hieroglyphic Texts
Source: PLoS One. 2014 Sep 25;9(9):e107982. doi: 10.1371/journal.pone.0107982 (PMC4177853; doi:10.1371/journal.pone.0107982)
Supplement: Table S4 — Calculated Ii and Gi* scores at all 25 km spatial lags. Significant values indicated in bold. (DOCX) [file pone.0107982.s004.docx]

**Table S4.** Calculated *I_i_* and *G_i_^*^* scores at all 25km spatial lags. Significant values indicated in bold.

| **ID** | **Site Name** | **Std. Ch'ahb'** | ***I_i_*(25)** | ***G_i_^*^*(25)** | ***I_i_*(50)** | ***G_i_^*^*(50)** | ***I_i_*(75)** | ***G_i_^*^*(75)** | ***I_i_*(100)** | ***G_i_^*^*(100)** | ***I_i_*(125)** | ***G_i_^*^*(125)** | ***I_i_*(150)** | ***G_i_^*^*(150)** | ***I_i_*(175)** | ***G_i_^*^*(175)** | ***I_i_*(200)** | ***G_i_^*^*(200)** |
| --- | --- | --- | --- | --- | --- | --- | --- | --- | --- | --- | --- | --- | --- | --- | --- | --- | --- | --- |
| 1 | Aguas Calientes | 0.000 | 0.276 | -0.410 | 0.590 | -0.643 | 0.644 | -0.550 | -0.546 | 0.245 | 0.547 | -0.420 | 0.794 | -0.594 | 0.532 | -0.493 | 1.003 | -0.897 |
| 2 | Aguateca | 0.000 | -0.082 | -0.087 | 0.141 | -0.230 | 0.455 | -0.448 | 1.053 | -0.763 | 0.078 | -0.140 | 0.342 | -0.315 | 0.218 | -0.258 | 0.911 | -0.826 |
| 3 | Altar de los Reyes | 0.000 | 0.157 | -0.568 | 0.785 | -1.014 | 1.570 | -1.428 | 2.533 | -1.760 | 3.161 | **-2.073** | 4.042 | **-2.522** | 4.137 | **-2.555** | 4.471 | **-2.920** |
| 4 | Altar de Sacrificios | 0.053 | 0.224 | -0.893 | 0.156 | -0.531 | -0.438 | 1.042 | -0.270 | 0.575 | -0.146 | 0.282 | 0.018 | -0.066 | 0.313 | -0.799 | 0.248 | -0.707 |
| 5 | Altun Ha | 0.000 | 0.000 | -0.399 | 0.314 | -0.701 | 0.471 | -0.815 | 0.628 | -0.918 | 1.519 | -1.296 | 2.629 | -1.799 | 3.119 | **-1.986** | 3.260 | **-2.034** |
| 6 | Arroyo de Piedra | 0.000 | 0.232 | -0.390 | 0.517 | -0.538 | 0.612 | -0.550 | -1.079 | 0.582 | 0.523 | -0.405 | 0.951 | -0.699 | 0.375 | -0.373 | 1.068 | -0.977 |
| 7 | Bejucal | 0.000 | 0.230 | -0.502 | 1.643 | -1.392 | 2.376 | -1.682 | 3.509 | **-2.202** | 4.340 | **-2.743** | 3.590 | **-2.488** | 2.226 | -1.902 | 2.540 | **-2.320** |
| 8 | Bonampak | 0.000 | -1.943 | **2.319** | -1.984 | **1.966** | -1.131 | 0.753 | -0.899 | 0.501 | -0.866 | 0.439 | -1.247 | 0.655 | 0.082 | -0.143 | 0.398 | -0.363 |
| 9 | Calakmul | 0.000 | 0.000 | -0.399 | 0.314 | -0.701 | 0.628 | -0.918 | 1.570 | -1.428 | 2.376 | -1.682 | 3.475 | **-2.232** | 4.042 | **-2.522** | 4.825 | **-3.308** |
| 10 | Cancuen | 0.250 | -0.376 | 0.396 | -0.200 | 0.380 | -0.861 | 0.013 | -1.201 | -0.085 | -3.080 | -0.575 | -0.035 | 0.216 | 3.436 | 1.155 | 3.675 | 1.367 |
| 11 | Caracol | 0.029 | 0.095 | -0.456 | 0.217 | -0.407 | 0.447 | -0.562 | 1.018 | -1.076 | 1.159 | -1.193 | 1.883 | **-1.981** | 2.330 | **-2.614** | 1.247 | -1.539 |
| 12 | Ceibal | 0.000 | -0.239 | 0.107 | 0.629 | -0.627 | 0.550 | -0.477 | 2.036 | -1.323 | 0.654 | -0.492 | 0.656 | -0.525 | 0.970 | -0.748 | 1.382 | -1.323 |
| 13 | Chinikiha | 0.000 | 0.157 | -0.568 | 0.249 | -0.475 | 0.208 | -0.366 | -1.463 | 0.981 | -1.306 | 0.841 | -0.677 | 0.351 | -0.913 | 0.473 | -0.525 | 0.220 |
| 14 | Chinkultic | 0.000 | -0.909 | 1.361 | -0.909 | 1.361 | -0.909 | 1.361 | -2.726 | **3.336** | -2.579 | **2.178** | -1.882 | 1.264 | -1.426 | 0.800 | -1.529 | 0.832 |
| 15 | Copan | 0.176 | -0.219 | 0.112 | -0.309 | -0.003 | 2.447 | **2.561** | 2.447 | **2.561** | 2.447 | **2.561** | 3.039 | **2.804** | 3.756 | **2.617** | 3.945 | **2.289** |
| 16 | Dos Pilas | 0.000 | 0.119 | -0.277 | 0.517 | -0.538 | 0.455 | -0.448 | -1.079 | 0.582 | 0.052 | -0.124 | 0.637 | -0.492 | 0.375 | -0.373 | 1.068 | -0.977 |
| 17 | El Cayo | 0.167 | -0.427 | -0.181 | 1.828 | 1.558 | 1.432 | 1.146 | 0.496 | 0.414 | 0.006 | 0.128 | 0.611 | 0.411 | 0.095 | 0.164 | -1.689 | -0.727 |
| 18 | El Chal | 0.000 | 0.000 | -0.399 | 0.336 | -0.443 | 1.626 | -1.104 | 1.831 | -1.187 | 2.145 | -1.383 | 2.962 | **-1.968** | 1.796 | -1.430 | 0.316 | -0.407 |
| 19 | El Chorro | 0.000 | 0.516 | -0.787 | 0.590 | -0.643 | -0.882 | 0.518 | -0.720 | 0.349 | -0.396 | 0.142 | 0.057 | -0.132 | 0.984 | -0.799 | 0.914 | -0.855 |
| 20 | El Kinel | 0.000 | 0.032 | -0.281 | -1.827 | 1.677 | -1.131 | 0.753 | -0.428 | 0.175 | 0.126 | -0.173 | -0.546 | 0.232 | 0.491 | -0.405 | 1.278 | -1.051 |
| 21 | El Palmar | 0.000 | 0.157 | -0.568 | 0.471 | -0.815 | 1.256 | -1.271 | 2.219 | -1.604 | 2.847 | -1.916 | 3.885 | **-2.438** | 3.980 | **-2.465** | 3.999 | **-2.476** |
| 22 | El Peru | 0.000 | 0.000 | -0.399 | 0.157 | -0.568 | 0.785 | -1.014 | 2.404 | -1.563 | 1.561 | -1.032 | 2.317 | -1.670 | 2.631 | -1.953 | 2.294 | **-2.108** |
| 23 | Itzan | 0.000 | 0.516 | -0.787 | 0.590 | -0.643 | -1.112 | 0.669 | -0.901 | 0.470 | -0.238 | 0.049 | 0.904 | -0.648 | 1.141 | -0.929 | 0.914 | -0.855 |
| 24 | Ixkun | 0.250 | -0.752 | 0.092 | -2.483 | -0.626 | -2.717 | -0.497 | -3.673 | -0.692 | -6.412 | -1.383 | -7.317 | -1.636 | -4.921 | -1.134 | -1.925 | -0.354 |
| 25 | Ixlu | 0.000 | 0.157 | -0.568 | 1.277 | -1.109 | 2.410 | -1.619 | 3.196 | **-1.994** | 3.087 | **-2.027** | 3.747 | **-2.635** | 2.069 | -1.721 | 2.226 | -1.902 |
| 26 | Ixtutz | 0.000 | -0.219 | 0.092 | 0.179 | -0.318 | 0.340 | -0.348 | 0.845 | -0.600 | 1.988 | -1.284 | 2.367 | -1.530 | 1.523 | -1.134 | 0.073 | -0.198 |
| 27 | Jimbal | 0.000 | 0.387 | -0.636 | 1.277 | -1.109 | 2.533 | -1.760 | 3.509 | **-2.202** | 4.295 | **-2.700** | 4.029 | **-2.825** | 4.020 | **-3.059** | 2.226 | -1.902 |
| 28 | Joloniel | 0.000 | 0.000 | -0.399 | -0.287 | 0.194 | -0.130 | -0.035 | 0.184 | -0.367 | 0.166 | -0.291 | -1.651 | 1.235 | -2.214 | 1.472 | -2.057 | 1.331 |
| 29 | Kendal | 0.000 | 0.000 | -0.399 | 0.000 | -0.399 | 0.000 | -0.399 | 0.271 | -0.430 | 0.676 | -0.562 | 2.004 | -1.311 | 2.716 | -1.709 | 1.120 | -0.764 |
| 30 | Kichpanha | 0.000 | 0.000 | -0.399 | 0.314 | -0.701 | 0.471 | -0.815 | 0.785 | -1.014 | 1.676 | -1.377 | 2.690 | -1.838 | 3.414 | **-2.194** | 3.528 | **-2.193** |
| 31 | Kuna Lacanha | 1.000 | **-1.974** | **3.289** | -4.126 | 1.558 | **-8.638** | 1.064 | **-24.434** | 0.021 | **-13.943** | 0.541 | **-13.097** | 0.558 | **-15.467** | 0.443 | **-28.510** | -0.183 |
| 32 | La Amelia | 0.000 | 0.276 | -0.410 | 0.590 | -0.643 | 0.487 | -0.451 | -0.703 | 0.348 | -0.081 | -0.045 | 0.166 | -0.196 | 0.532 | -0.493 | 0.914 | -0.855 |
| 33 | La Corona | 0.000 | 0.000 | -0.399 | 0.157 | -0.568 | 0.314 | -0.701 | 1.270 | -1.072 | 1.215 | -0.817 | 2.262 | -1.467 | 2.823 | **-2.047** | 2.856 | **-2.265** |
| 34 | La Mar | 0.000 | -0.174 | 0.026 | -1.960 | 1.810 | -1.489 | 1.146 | -1.107 | 0.714 | -1.029 | 0.563 | -1.310 | 0.708 | -0.368 | 0.126 | -0.117 | -0.024 |
| 35 | La Milpa | 0.000 | 0.000 | -0.399 | 0.471 | -0.815 | 1.413 | -1.350 | 2.533 | -1.760 | 3.257 | **-2.114** | 3.885 | **-2.438** | 3.685 | **-2.285** | 3.501 | **-2.197** |
| 36 | La Pasadita | 0.000 | -0.324 | 0.248 | -1.803 | 1.558 | -1.332 | 0.972 | -0.503 | 0.242 | -0.046 | -0.069 | -0.400 | 0.145 | -0.274 | 0.070 | 0.712 | -0.587 |
| 37 | La Rejolla | 0.000 | 0.252 | -0.609 | 0.610 | -0.634 | 0.748 | -0.592 | 1.774 | -1.165 | 2.162 | -1.380 | 3.558 | **-2.399** | 3.937 | **-2.763** | 1.994 | -1.539 |
| 38 | La Sufricaya | 0.000 | 0.106 | -0.341 | 1.277 | -1.109 | 2.629 | -1.799 | 3.038 | -1.949 | 3.842 | **-2.380** | 3.732 | **-2.430** | 3.872 | **-2.676** | 4.061 | **-2.954** |
| 39 | Lamanai | 0.000 | 0.000 | -0.399 | 0.471 | -0.815 | 0.785 | -1.014 | 1.362 | -1.212 | 2.629 | -1.799 | 3.571 | **-2.274** | 3.528 | **-2.193** | 3.417 | **-2.130** |
| 40 | Los Higos | 1.000 | 0.000 | **5.018** | **-2.783** | **2.609** | -0.028 | **2.561** | -0.028 | **2.561** | 3.334 | **2.410** | **7.043** | **2.399** | **12.548** | **2.372** | 4.377 | 1.567 |
| 41 | Lubaantun | 0.000 | -0.876 | 1.071 | -0.719 | 0.730 | -0.529 | 0.334 | 0.256 | -0.310 | -1.841 | 1.074 | -0.751 | 0.354 | 0.173 | -0.203 | 1.272 | -0.949 |
| 42 | Machaquila | 0.182 | -0.231 | 0.133 | -0.249 | 0.052 | -1.146 | -0.378 | -2.046 | -0.720 | -3.258 | -1.187 | -1.422 | -0.461 | -2.088 | -0.804 | -0.020 | 0.173 |
| 43 | Motul de San Jose | 0.000 | 0.157 | -0.568 | 0.701 | -0.861 | 1.509 | -1.124 | 3.555 | **-2.216** | 3.838 | **-2.474** | 1.441 | -1.119 | 1.912 | -1.555 | 2.540 | **-2.320** |
| 44 | Naachtun | 0.000 | 0.000 | -0.399 | 1.099 | -1.189 | 1.957 | -1.548 | 2.533 | -1.760 | 3.632 | **-2.312** | 3.980 | **-2.465** | 4.640 | **-2.997** | 4.419 | **-3.352** |
| 45 | Naj Tunich | 0.000 | -0.424 | 0.484 | -0.781 | 0.627 | -0.445 | 0.230 | -0.088 | -0.046 | 1.223 | -0.821 | 0.235 | -0.238 | 0.330 | -0.302 | 1.775 | -1.415 |
| 46 | Nakum | 0.000 | 0.178 | -0.361 | 1.748 | -1.363 | 2.253 | -1.537 | 3.038 | -1.949 | 2.947 | -1.873 | 3.872 | **-2.676** | 3.904 | **-2.789** | 2.069 | -1.721 |
| 47 | Naranjo | 0.171 | -1.042 | -0.621 | -2.724 | -1.319 | -3.267 | -1.455 | -4.725 | **-2.033** | -4.923 | **-2.096** | -5.565 | **-2.607** | -5.625 | **-2.676** | -6.083 | **-3.130** |
| 48 | Nim Li Punit | 0.286 | -0.452 | 0.534 | 0.318 | 0.730 | -0.231 | 0.334 | -2.490 | -0.310 | -3.672 | -0.532 | -1.183 | 0.024 | -2.575 | -0.272 | -5.868 | -1.070 |
| 49 | Pacbitun | 0.000 | -0.051 | -0.157 | 0.829 | -0.887 | 1.468 | -1.116 | 2.743 | -1.749 | 2.873 | -1.803 | 3.261 | **-2.086** | 3.872 | **-2.676** | 3.872 | **-2.676** |
| 50 | Palenque | 0.115 | 0.000 | 0.226 | -0.066 | -0.035 | -0.243 | -0.367 | -0.234 | -0.291 | -0.322 | -0.413 | 0.689 | 0.981 | 1.026 | 1.331 | 0.645 | 0.793 |
| 51 | Piedras Negras | 0.063 | -0.006 | 0.026 | -0.253 | 1.558 | -0.205 | 1.146 | -0.124 | 0.581 | -0.022 | 0.080 | -0.107 | 0.411 | -0.036 | 0.126 | 0.074 | -0.314 |
| 52 | Pomona | 0.000 | 0.157 | -0.568 | 0.181 | -0.503 | 0.208 | -0.366 | -1.421 | 1.009 | -1.306 | 0.841 | -0.992 | 0.584 | -1.070 | 0.576 | -0.368 | 0.126 |
| 53 | Pusilha | 0.273 | -0.849 | 0.164 | 0.373 | 0.730 | -0.142 | 0.334 | 0.475 | 0.451 | 2.131 | 0.751 | 0.204 | 0.298 | -2.039 | -0.203 | -4.160 | -0.720 |
| 54 | Quirigua | 0.043 | 0.000 | -0.163 | -0.834 | **2.561** | -0.834 | **2.561** | -1.008 | **2.804** | -1.065 | **2.399** | -1.274 | **2.289** | -1.107 | 1.846 | -0.947 | 1.418 |
| 55 | Río Amarillo | 0.250 | -0.219 | 0.112 | -2.129 | **2.561** | -2.129 | **2.561** | -2.129 | **2.561** | -2.129 | **2.561** | -2.397 | **2.410** | -3.067 | **2.617** | -3.203 | **2.289** |
| 56 | Sacchana | 0.000 | -0.909 | 1.361 | -0.909 | 1.361 | -0.909 | 1.361 | -1.817 | 0.885 | **7.793** | **2.427** | **6.543** | 1.577 | 2.083 | 0.800 | 3.588 | 0.933 |
| 57 | Sacul | 0.500 | -0.219 | 0.092 | 0.504 | -0.626 | 0.288 | -0.300 | 0.688 | -0.507 | 1.787 | -1.158 | 2.838 | -1.854 | 1.805 | -1.324 | 0.271 | -0.354 |
| 58 | San Bartolo | 0.000 | 0.157 | -0.568 | 1.120 | -1.019 | 2.219 | -1.604 | 3.257 | **-2.114** | 3.980 | **-2.465** | 3.925 | **-2.443** | 4.248 | **-2.883** | 3.863 | **-2.876** |
| 59 | Tamarindito | 0.000 | -3.321 | -0.390 | -4.327 | -0.538 | -4.662 | -0.550 | -6.216 | -0.763 | -4.683 | -0.463 | -5.857 | -0.699 | -3.274 | -0.258 | -6.269 | -0.977 |
| 60 | Tayasal | 0.333 | 0.314 | -0.701 | 0.858 | -0.960 | 1.530 | -1.054 | 3.241 | **-2.022** | 3.100 | **-2.001** | 1.441 | -1.119 | 1.912 | -1.555 | 2.540 | **-2.320** |
| 61 | Tikal | 0.000 | -0.421 | -0.754 | -0.900 | -1.196 | -1.405 | -1.682 | -1.929 | **-2.117** | -2.520 | **-2.812** | -2.226 | **-2.789** | -1.240 | -1.721 | -1.325 | -1.902 |
| 62 | Toniná | 0.132 | 0.000 | -0.399 | 0.000 | -0.399 | 0.068 | -0.335 | -2.560 | 1.918 | -2.214 | 1.472 | -2.214 | 1.472 | -1.698 | 1.024 | -1.467 | 0.811 |
| 63 | Tortuguero | 0.000 | 0.000 | 0.504 | -0.086 | 0.194 | -0.086 | 0.194 | -0.482 | -0.215 | -0.681 | -0.367 | -0.659 | -0.291 | 1.636 | 1.235 | 2.347 | 1.472 |
| 64 | Tres Islas | 0.167 | -0.606 | 0.669 | -0.532 | 0.355 | 0.298 | -0.342 | 0.864 | -0.668 | 1.864 | -1.206 | 0.267 | -0.260 | 0.720 | -0.574 | -1.597 | 1.113 |
| 65 | Uaxactun | 0.000 | 0.230 | -0.502 | 1.277 | -1.109 | 2.376 | -1.682 | 3.352 | **-2.117** | 3.353 | **-2.089** | 4.673 | **-3.118** | 4.044 | **-3.157** | 2.226 | -1.902 |
| 66 | Ucanal | 0.000 | 0.157 | -0.568 | 1.081 | -0.930 | 2.253 | -1.537 | 1.617 | -1.076 | 2.572 | -1.655 | 3.558 | **-2.399** | 3.904 | **-2.789** | 0.135 | -0.233 |
| 67 | Uolantun | 0.000 | 0.387 | -0.636 | 1.748 | -1.363 | 2.253 | -1.537 | 3.352 | **-2.117** | 4.000 | **-2.554** | 3.872 | **-2.676** | 4.218 | **-3.130** | 2.226 | -1.902 |
| 68 | Uxul | 0.000 | 0.000 | -0.399 | 0.314 | -0.701 | 1.413 | -1.350 | 2.428 | -1.775 | 2.847 | -1.916 | 3.632 | **-2.312** | 4.982 | **-3.468** | 3.478 | **-2.662** |
| 69 | Xultun | 0.000 | 0.314 | -0.701 | 1.277 | -1.109 | 2.376 | -1.682 | 3.414 | **-2.194** | 3.980 | **-2.465** | 3.529 | **-2.224** | 4.248 | **-2.883** | 4.020 | **-3.059** |
| 70 | Xunantunich | 0.000 | -0.051 | -0.157 | 0.986 | -0.983 | 1.782 | -1.287 | 2.900 | -1.836 | 3.030 | -1.899 | 3.575 | **-2.311** | 3.872 | **-2.676** | 3.904 | **-2.789** |
| 71 | Yaxchilan | 0.000 | -0.376 | -0.449 | 1.215 | 1.558 | 0.678 | 0.753 | 0.242 | 0.280 | -0.386 | -0.221 | 0.462 | 0.419 | -0.493 | -0.304 | -1.248 | -1.051 |
| 72 | Yaxha | 0.133 | 0.106 | -0.341 | 1.687 | -1.319 | 2.253 | -1.537 | 2.807 | -1.798 | 3.261 | **-2.086** | 3.872 | **-2.676** | 3.904 | **-2.789** | 2.069 | -1.721 |
